# Association of sepsis-induced cardiomyopathy and mortality: a systematic review and meta-analysis

**DOI:** 10.1186/s13613-022-01089-3

**Published:** 2022-12-13

**Authors:** Yu-Min Lin, Mei-Chuan Lee, Han Siong Toh, Wei-Ting Chang, Sih-Yao Chen, Fang-Hsiu Kuo, Hsin-Ju Tang, Yi-Ming Hua, Dongmei Wei, Jesus Melgarejo, Zhen-Yu Zhang, Chia-Te Liao

**Affiliations:** 1 Division of Cardiology, Department of Internal Medicine, Chi Mei Medical Centre, No.901, Zhonghua Rd. Yongkang Dist., 71004 Tainan, Taiwan; 2 Department of Pharmacy, Chi Mei Medical Centre, Tainan, Taiwan; 3 Department of Public Health, College of Medicine, National Cheng Kung University, Tainan, Taiwan; 4 Department of Intensive Care Medicine, Chi Mei Medical Centre, Tainan, Taiwan; 5 Institute of Clinical Medicine, College of Medicine, National Cheng Kung University, Tainan, Taiwan; 6 Department of Health and Nutrition, Chia Nan University of Pharmacy and Science, Tainan, Taiwan; 7 Department of Biotechnology, Southern Taiwan University of Science and Technology, Tainan, Taiwan; 8 Department of Nursing, Chang Gung University of Science and Technology, Chiayi, Taiwan; 9 Studies Coordinating Centre, Research Unit Hypertension and Cardiovascular Epidemiology, KU Louvain Department of Cardiovascular Sciences, University of Leuven, Leuven, Belgium

**Keywords:** Sepsis, Ventricular dysfunction, Cardiomyopathies, Mortality

## Abstract

**Background:**

The implication of sepsis-induced cardiomyopathy (SIC) to prognosis is controversial, and its association with mortality at different stages remains unclear. We conducted a systematic review and meta-analysis to understand the association between SIC and mortality in septic patients.

**Methods:**

We searched and appraised observational studies regarding the mortality related to SIC among septic patients in PubMed and Embase from inception until 8 July 2021. Outcomes comprised in-hospital and 1-month mortality. We adopted the random-effects model to examine the mortality risk ratio in patients with and without SIC. Meta-regression, subgroup, and sensitivity analyses were applied to examine the outcome’s heterogeneity.

**Results:**

Our results, including 20 studies and 4,410 septic patients, demonstrated that SIC was non-statistically associated with increased in-hospital mortality, compared to non-SIC (RR 1.28, [0.96–1.71]; *p* = 0.09), but the association was statistically significant in patients with the hospital stay lengths longer than 10 days (RR 1.40, [1.02–1.93]; *p* = 0.04). Besides, SIC was significantly associated with a higher risk of 1-month mortality (RR 1.47, [1.17–1.86]; *p* < 0.01). Among SIC patients, right ventricular dysfunction was significantly associated with increased 1-month mortality (RR 1.72, [1.27–2.34]; *p* < 0.01), while left ventricular dysfunction was not (RR 1.33, [0.87–2.02]; *p* = 0.18).

**Conclusions:**

With higher in-hospital mortality in those hospitalized longer than 10 days and 1-month mortality, our findings imply that SIC might continue influencing the host’s system even after recovery from cardiomyopathy. Besides, right ventricular dysfunction might play a crucial role in SIC-related mortality, and timely biventricular assessment is vital in managing septic patients.

**Supplementary Information:**

The online version contains supplementary material available at 10.1186/s13613-022-01089-3.

## Background

Sepsis is a dysregulated immune response due to infection, leading to life-threatening organ dysfunction, e.g., respiratory, renal, immunological, digestive, neurological, and cardiovascular organs [1]. The prevalence of cardiovascular dysfunction caused by sepsis may reach up to 50% [2], and the symptoms may comprise vasodilatory shock, myocardial injury, arrhythmia, and sepsis-induced cardiomyopathy (SIC) [3]. The exact mechanism is still not well understood, any may include vasoplegia, impaired myocardial circulation, direct myocardial depression, and mitochondrial dysfunction [4, 5].

SIC is an increasingly recognized condition of transient myocardial impairment in septic patients [2, 6]. The growing evidence shows that apart from left ventricular (LV) systolic dysfunction (LVSD), SIC is further associated with LV diastolic and right ventricular dysfunction due to hypoxemia, pulmonary vessel vasoconstriction and remodeling [7, 8]. Despite the findings, the relationship between SIC and mortality remains debatable [9, 10]. Sevilla Berrios RA et al. showed that the LV systolic function during sepsis was not significantly related to mortality [11]. In contrast, Sanfilippo F et al. presented that LV diastolic dysfunction (LVDD) was associated with mortality [12]. Vallabhajosyula S et al. demonstrated that right ventricular (RV) dysfunction was related to short-term and long-term mortality [13]. The primary outcome of these meta-analyses focused on the mortality at the acute stage, i.e., intensive care unit and in-hospital stay, and at the subacute stage, i.e., 1 month after admission [11, 12]. However, the impact of overall SIC on mortality at different stages remains ambiguous. Besides, previous studies utilized pulmonary artery catheterization, which was itself associated with mortality in septic patients, to evaluate RV dysfunction [13]. With the improvement in technology and skills, echocardiography is more widely used nowadays to evaluate myocardial tissue properties or strain to detect more subtle myocardial function abnormalities [14, 15].

Accordingly, this study aimed to conduct a systematic review and meta-analysis to examine the association between SIC and mortality at different stages and investigate the impact of LV and RV dysfunction on mortality in septic patients.

## Methods

### Data sources and searches

Our study utilized Embase and PubMed as our bibliographic databases. Included articles were published up to 8 July 2021. Free texts and controlled synonymous vocabularies for sepsis, heart dysfunction, and mortality were defined. (Additional file 1: Appendix Table S1). Furthermore, we manually conducted a cross-reference search of relevant articles.

### Study selection

We included articles that examined the association between SIC and mortality in patients with sepsis. According to Beesley SJ et al., SIC is defined as a transient systolic or diastolic dysfunction of LV or RV due to sepsis; the dysfunction caused by coronary artery diseases is commonly excluded from the SIC [2]. The definition covers most conditions and is not limited to specific evaluation tools. We excluded the studies if their outcomes were out of our interest and lacked detailed mortality numbers, e.g., developing a mortality prediction model for patients with SIC, investigating the deviant cut-off values of diagnostic tools for SIC, and examining the characteristic of echocardiographic parameters in mortality patients. We also excluded study protocols, conference abstracts, pediatrics, obstetrics, and animal model articles. Only papers written in English were included.

In our study, the exposure of interest was transient myocardial dysfunction caused by sepsis, and the myocardial dysfunction improved after sepsis. The diagnostic tools were examined by two reviewers (YML and MCL), and all values of the tools regarding myocardial function were verified in the enrolled articles. The primary outcome of our study was mortality after admission, including in-hospital and 1-month mortality. Follow-up duration and the number of subjects lost to follow-up were recorded and analyzed.

### Data extraction and quality assessment

Two reviewers (YML and MCL) extracted the publication types, inclusion and exclusion criteria, population characteristics, average age, subject numbers, SIC definition, time for operating echocardiography, mortality, percentage of mechanical ventilation users, ratio of septic shock, acute physiology and chronic health evaluation (APACHE) score, sequential organ failure assessment (SOFA) score, length of hospital or intensive care unit stay from the included citations. We contacted the authors by e-mail if the data were insufficient. The two reviewers (YML and MCL) independently appraised each study with three domains based on the Newcastle–Ottawa scale (Additional file 1: Appendix Table S2), including selection, comparability, and outcome. The third author (CTL) was consulted if there were any disagreements.

### Data synthesis and meta-analysis

Our systematic review and meta-analyses followed the Preferred Reporting Items for Systemic Reviews and Meta-Analyses (PRISMA) guidelines [16]. Data were analyzed using RevMan 5.4. and SPSS version 28.0 (SPSS Inc., Chicago, IL). Since the included studies had various risk estimate methods (i.e., risk ratios (RRs), hazard ratios and odds ratios), with or without risk estimate adjustment, and lacked consistency, we extracted the original number of septic patients with SIC and non-SIC and the number of mortalities from the selected studies. The mortality outcomes were analyzed using RRs as the summary statistics, and the precision levels of the effect sizes were reported as 95% confidence intervals (95% CIs). A pooled estimate of the RR was computed using the DerSimonian and Laird random-effects model to minimize the effect of the subject numbers in different articles [17]. Besides, the *I*^2^ statistics was used to evaluate heterogeneity. Low, moderate, or high heterogeneity was defined as *I*^2^ ≤ 25%, 25% < *I*^2^ < 75%, and *I*^2^ ≥ 75%, respectively.

### Subgroup and sensitivity analysis

We carried out subgroup analyses to examine the heterogeneity of the outcomes. First, we divided in-hospital mortality into early and late acute-stage mortality to account for the diversity of hospital stay length across citations. Since the myocardial function commonly recovered from SIC within 10 days according to previous studies, we used 10 days as the cut-off point to assess the heterogeneity [6]. Second, SIC was divided into LV and RV dysfunction to evaluate the heterogeneity of individual impacts on 1-month and in-hospital mortality among septic patients. Third, the other subgroup analyses were based on the definition of sepsis (sepsis II and III), the indication of echocardiography (by protocol and clinical needs), the operation timing (Days 1, 2, 3), different cut-off values of SIC diagnosis [LV ejection fraction (LVEF) < 50%, LVEF reduction > 10%, E/e’ > 15, RV S’ < 15 cm/s, and tricuspid annular plane systolic excursion (TAPSE) < 16 mm], study’s appraisal quality, and with or without risk estimate adjustment. Apart from subgroup analyses, a random-effects meta-regression with Egger's test was carried out to examine the impact of individual variables on the outcomes and heterogeneity.

Sensitivity analyses were also conducted to evaluate the heterogeneity or the range of result uncertainty. A one-by-one exclusion method was performed to assess the influence of each article on our results. In our selected studies, we noticed that two studies (Vallabhajosyula, S et al.) had the same hospital units and study periods, and another two (Innocenti F et al.) had partial duplicate patients. Despite the potential duplicate patients, the studies focused on different ventricular dysfunctions and lacked individual-level patient information, so we remained the studies in our analyses and performed the sensitivity analyses to evaluate the range of result uncertainty. Moreover, other sensitivity analyses were carried out to evaluate the range of the outcome’s uncertainty due to unclear data definition or overlap.

### Role of the funding source

Our research was funded by Chi Mei Medical Centre (CMFHR11153) and Internal Funds KU Leuven (STG-18-00379). The funding source had no part in the design or conduct of this review.

## Results

### Characteristics of included studies and population

**Fig. 1:**
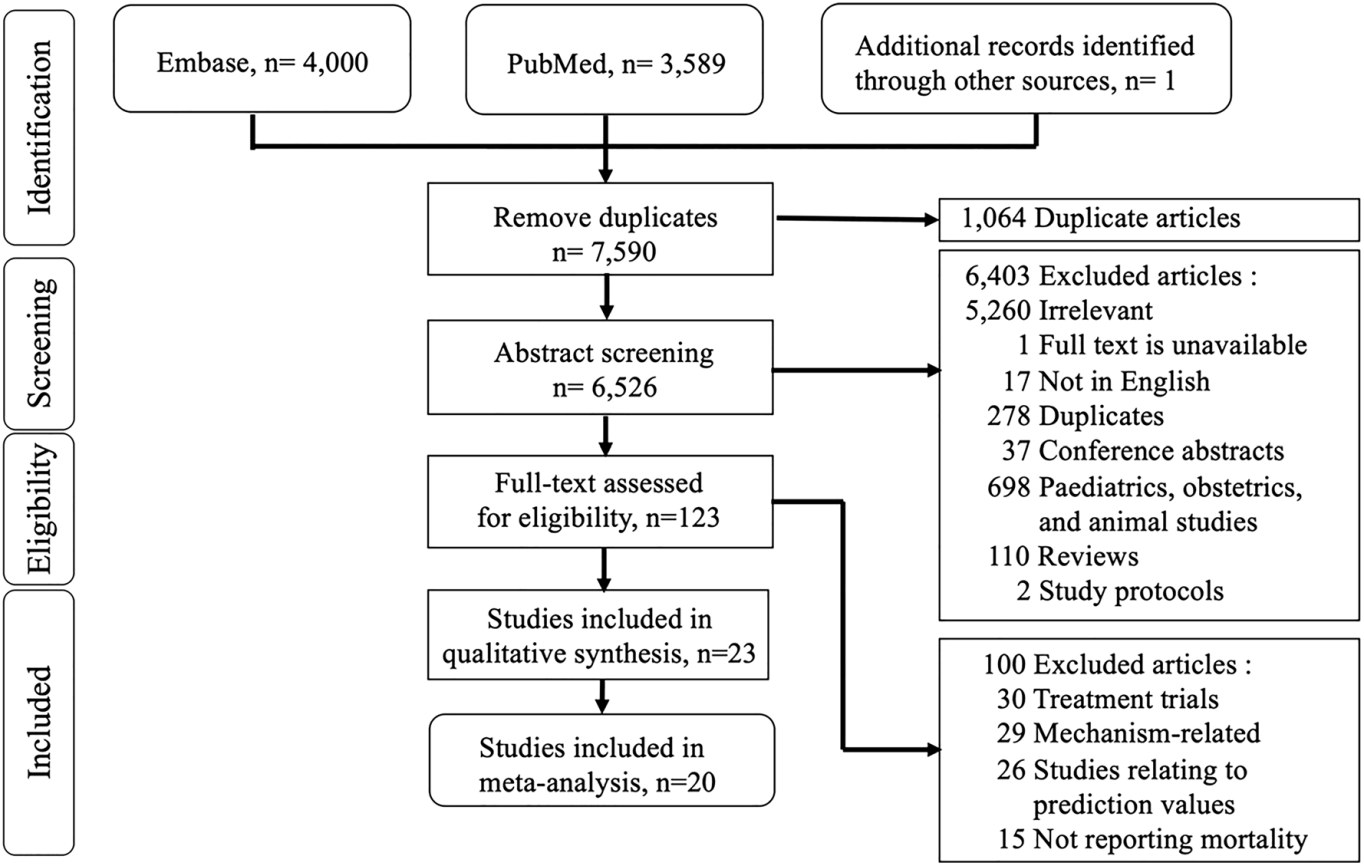
Literature search flow diagram according to the Preferred Reporting Items for Systemic Reviews and Meta-Analyses (PRISMA) guidelines

Figure 1 presents our flow chart of the literature search. Our search strategy found 7,590 articles: 4,000 articles from Embase, 3,589 articles from PubMed, and one article from the manual reference search of related papers [18]. After removing 1,064 duplicates, 6,526 articles were suitable for abstract screening. After excluding irrelevant articles, conference abstracts, pediatrics, obstetrics, and animal studies, there were 123 studies with full-text access. Then, 100 articles related to therapy, mechanism, or prediction value, and lacked mortality outcomes were excluded. Subsequently, 23 studies were enrolled in our qualitative synthesis, including 21 cohort studies and two case–control studies. We removed three articles due to a lack of analyzable data [8, 19, 20]. Finally, the meta-analysis pooled 20 studies, including 4,410 septic patients [10, 18, 21–38]. The mean age of the study population ranged from 38.8 to 77 years, and the mean length of hospital stay in all included studies ranged from 4.9 days to 43 days [25, 33]. These studies did not report the sex ratio, and the prevalence of cardiovascular comorbidities was inconsistently informed. (Table 1).

**Table 1:** Main characteristics of included studies

Study (year)	Study design	SIC definition	Patient number (SIC, non-SIC)	Time of echocardiogram	Age(years)	Cardiovascular comorbidity (%)	APACHE score	Hospital LOS (days)	Inotropic agents (%)	Mechanical ventilation (%)	Septic shock (%)	Outcome
Artucio (1989) [^21^]	Cohort study	LVSD: PEP/LVET > 42%	33, 16	Day 1	38.8 ± 2.3	Excluded septic shock	NA	NA	0	63	0	Mortality
Charpentier (2004) [^10^]	Cohort study	LVSD: FAC < 50%	15, 19	Day 2	56 ± 2.7	Excluded CAD	NA	NA	SIC 66.6 non-SIC 73.6	SIC 60 Non-SIC 47	74	One-month mortality
Pulido (2012) [^22^]	Cohort study	LVSD: LVEF < 50% or LVDD: E/e' > 15 or RVD: s' < 15 cm/s	68, 38	Day 1	Survivors 63 ± 15 Non-survivors 69 ± 15	Excluded CAD	APACHE iii Survivors 83.8 ± 28 Non-survivors 96.3 ± 31	NA	LVSD 21 LVDD 20 RVD 30 non-SIC 16	LVSD 86 LVDD 62 RVD 82 Non-SIC 74	NA	One-month mortality One-year mortality
Landesberg (2012) [^8^]	Cohort study	LVDD: e' < 8 cm/s	131, 95	Day 2	Survivors 56 ± 21 Non-survivors 70 ± 17	Excluded CAD	APACHE ii Survivors 18.8 ± 6.5 Non-survivors 24.5 ± 7.6	NA	EPI 21 DA / DBA 8	100	62	In-hospital mortality
Wilhelm (2013) [^19^]	Cohort study	LVSD: ACP ≤ 80%	NA	Day 1	Sepsis 68 (54–76) Severe sepsis 71 (57–79) Septic shock 69 (59–77)	NA	APACHE ii Sepsis 14 (10–18) Severe sepsis 17 (13–23) Septic shock 23 (18–29)	NA	NA	NA	19	One-month mortality
Mourad (2013) [^23^]	Case–control study	LVDD: e' ≤ 8 cm/s	33, 39	Day 2	58 (49–66)	CAD 6.9%	NA	NA	NA	SIC 54.5 Non-SIC 30.7	100	ICU mortality
Prabhu (2015) [^24^]	Case–control study	LVSD: LVEF < 50%	18, 48	NA	53.71 ± 16.76	Excluded CAD	APACHE iii Survivor 70.65 ± 13.27 Nonsurvivor 89.34 ± 15.41	9.76 ± 4.21	DA 19.7%	NA	100	Mortality
Sato (2016) [25]	Cohort study	LVSD: LVEF < 50% Decreased ≥ 10% EF Recovered within 2 weeks	29, 181	Day 1	SIC 69 (57–79) Non-SIC 77 (65–85)	CAD 12 HF 20	APACHE ii SIC 27 non-SIC 21	SIC 43 non-SIC 26	AVP SIC 37.9 non-SIC 13.3	NA	51	In-hospital mortality One-month mortality
Vallabhajosyula (2016) [28]	Cohort study	LVSD: LVEF < 50% or LVDD: E/e' > 15	LVSD (17, 41) LVDD (11, 47)	Day 1	68.0 (54.8–76.0)	CAD 19 AF 24	APACHE iii 86.0 (72.0—115.5)	LVSD: 14.7 (9.2–20.5) non LVSD: 23.0 (8.0–34.4) LVDD: 23.1 (8.2–31.8) non LVDD: 15.9 (9.0–31.0)	NA	100	NA	In-hospital mortality
Vallabhajosyula (2017) [26]	Cohort study	RVD: TAPSE < 16 mm	214, 174	Day 3	IRVD 65.6 (55.2–77.5) RVD/LVD 69.3 (55.3–77.4) No RVD 64.7 (53.4–74.7)	CAD 14.4	APACHE iii IRVD 85.5 (68.3–110) RVD/LVD 84.0 (69.0–104) No RVD 81.0 (66.0–105)	IRVD 9.3 (5.8–19.4) RVD/LVD 8.5 (6.0–14.4) No RVD 9.8 (6.1–16.6)	NA	IRVD 67.0 RVD/LVD 50.9 No RVD 50.6	IRVD 80.0 RVD/LVD 71.9 No RVD 68.4	In-hospital mortality One-year mortality
Narváez (2017) [29]	Cohort study	LVSD: LVEF < 50%	13, 44	Day 1	SIC 54.5 ± 12.9 Non-SIC 64.4 ± 16.6	Excluded CAD	APACHE ii SIC 20.8 ± 8.7 Non-SIC 19.6 ± 8.1	SIC 22.66 ± 21.10 Non-SIC 24.24 ± 20.85	DBA 31.5	SIC 50.0 Non-SIC 43.2	SIC 76.9 Non-SIC 68.2%	ICU mortality In-hospital mortality
Boissier (2017) [20]	Cohort study	LVSD: LVEF < 45%	29, 103	Day 1	*Hypokinesia 64 (50–71) Normokinesia 65 (54–75) Hyperkinesia 63 (50–75)	Excluded CAD	NA	NA	EPI 4.5 DBA 10.6	80	100	ICU mortality In-hospital mortality
Vallabhajosyula (2018) [30]	Cohort study	LVSD: LVEF < 50%	206, 228	Day 3	SIC 68.0 (55.7–78.1) Non-SIC 65.6 (55.3–77.1)	CAD 20 AF 10	APACHE iii SIC 85 (70–109) Non-SIC 82 (68–103)	SIC 8.7 (6–15) Non-SIC 9.1 (6–18.8)	NA	SIC 52.4 Non-SIC 55.7	SIC 72.8 Non-SIC 70.6	In-hospital mortality Two-year mortality
Jeong (2018) [27]	Cohort study	LVSD: LVEF < 50%, Decreased ≥ 10% EF and Recovered within 2 weeks	25, 273	Day 1	SIC 70.6 ± 14.7 Non-SIC 71.8 ± 11.8	HF 12 CAD 5.3 AF 8	NA	SIC 20.8 ± 26.0 Non-SIC 30.2 ± 35.0	44.2%	NA	NA	In-hospital mortality
Rahasto (2019) [31]	Cohort study	LVSD: abnormal LVEF undefined	15, 87	Day 1	48 ± 18	Excluded CAD	NA	NA	NA	NA	100	Ten days mortality
Lahham (2020) [33]	Cohort study	RVD: TAPSE < 16 mm	8, 16	NA	56 ± 18	Excluded CAD	NA	4.91 ± 3.08	NA	0	NA	In-hospital mortality
Shin (2020) [34]	Cohort study	LVSD: LVEF < 50%	36, 330	Day 2	†Reduced 75.4 ± 12.5 Normal 72.3 ± 13.5 Hyperdynamic 72.8 ± 13.0	Exclude CAD	NA	†Reduced 23.2 ± 22.7 Normal 25.5 ± 22.2 Hyperdynamic 17.9 ± 14.0	NA	NA	NA	In-hospital mortality
Innocenti (Mar.2020) [36]	Cohort study	RVD: TAPSE < 16 mm	85, 167	Day 1	SIC 77 ± 13 Non-SIC 71 ± 15	CAD 19	APACHE ii SIC 19 ± 5 Non-SIC 18 ± 5	NA	NA	0	40	Seven days mortality One-month mortality
Song (2020) [35]	Cohort study	LVSD: LVEF < 50%, Decreased ≥ 10% LVEF and Recovered within 2 weeks	49, 259	Day 2	SIC 65.1 ± 11.2 Non-SIC 64.6 ± 15.0	Excluded patients without baseline echocardiography	APACHE ii SIC 26.4 ± 9.3 Non-SIC 24.6 ± 8.6	NA	SIC 100 Non-SIC 94.9	SIC 73.5 Non-SIC 63.3	SIC 100 Non-SIC 95	ICU mortality In-hospital mortality One-month mortality
Kim (2020) [32]	Cohort study	LVSD: LVEF < 50% or LVDD: E/e' > 15 or RVD: TAPSE < 16 mm	270, 508	Day 3	SIC 67.0 (58.0–75.0) Non-SIC 68.0 (55.8–76.0)	Excluded CAD Excluded Pt with normal troponin I	NA	NA	NA	SIC 50 Non-SIC 43.2	100	One-month mortality
Lanspa (2020) [18]	Cohort study	RVD: FAC < 35% or TAPSE < 16 mm	181, 194	Day 1	SIC 64.7 ± 16.0 Non-SIC 60.9 ± 16.5	NA	APACHE ii SIC 27.1 ± 10.1 Non-SIC 24.4 ± 10.1	NA	NA	SIC 28.7 Non-SIC 24.2	NA	One-month mortality
Innocenti (Oct.2020) [37]	Cohort study	RVD: TAPSE < 16 mm	120, 234	Day 1	Shock 74 ± 12 Non-shock 74 ± 14	Excluded MV patients	NA	NA	NA	0%	40	Seven days mortality One-month mortality
Chayakul (2020) [38]	Cohort study	LVSD: LVEF < 50%	24, 51	Day 1	SIC 73.1 ± 17.4 Non-SIC 65.8 ± 16.5	HFpEF 18.6	APACHE ii SIC 25.7 ± 6.7 Non-SIC 22.3 ± 5.6	NA	EPI 9.3 DA 6.6 DBA 6.6	SIC 50.0 Non-SIC 17.6	SIC 62.5 Non-SIC 68.6	In-hospital mortality

*SIC* Sepsis-induced cardiomyopathy, *LVSD* Left ventricular systolic dysfunction, *LVDD* Left ventricular diastolic dysfunction, *RVD* right ventricular dysfunction, *PEP/LVET* Pre-ejection period and left ventricular ejection time ratio, *FAC* Fractional area change, *LVEF* Left ventricular ejection fraction, *ACP* Afterload-related cardiac performance, *CAD* Coronary artery disease, *HF* Heart failure, *APACHE* Acute physiology and chronic health evaluation score, *ICU* Intensive care unit, *LOS* Length of stay, *EPI* Epinephrine, *DA* Dopamine, *DBA* Dobutamine, *AVP* Vasopressin, *NA* Not applicable, *TAPSE* Tricuspid annular plane systolic excursion, *IRVD* Isolated right ventricular dysfunction, *LVD* Left ventricular dysfunction, *AF* Atrial fibrillation, *Pt* Patient, *MV* Mechanical ventilation, *HFpEF* Heart failure with preserved ejection fraction

^*^Hypokinesia, normokinesia and hyperkinesia were defined as left ventricular ejection fraction < 45%, 45–60%, and > 60%, respectively

^†^Reduced, normal, hyperdynamic were defined as left ventricular ejection fraction < 50%, 50–70%, and > 70%, respectively

Table 1 shows the enrolled 23 articles in our qualitative synthesis. Most studies detected myocardial dysfunction with echocardiography, and one did so with afterload-related cardiac performance [19]. Among the studies using an echocardiogram, 17 defined SIC with LV dysfunction [8, 10, 20–25, 27–32, 34, 35, 38], and seven included RV dysfunction as well [18, 22, 26, 33, 36–38], Among the 17 LV dysfunction studies, 15 showed LVSD, and 12 of them defined abnormal LVSD as LVEF < 50%. One study described LVSD as pre-ejection period and LV ejection time ratio greater than 42% [21], and another presented LVSD with the ratio of fractional area change smaller than 50% [10]. One study did not mention the LVEF value in the article [31]. In the 15 LVSD studies, three defined SIC using 2 weeks reversibility [25, 27, 35]. Five studies evaluated LVDD using the definition of E/e’ greater than 15 [22, 28, 32], or e' smaller than 8 cm/s [8, 23]. For seven studies regarding RV dysfunction, one study defined RV dysfunction as s’ smaller than 15 cm/s [22], and six studies used TASPE smaller than 16 mm as the cut-off point [18, 26, 32, 33, 36, 37]. In the recruited studies, Pulido JN et al. showed that patients with acute respiratory distress syndrome (ARDS) constituted 16% and 18% of the normal and abnormal RV function groups, and there were 28.2% and 30.8% in the study of Vallabhajosyula S et al. [22, 26]. Notably, the ARDS percentages in these two groups were not significantly different. The other research did not provide the prevalence of ARDS.

Ten studies excluded patients with a history of coronary artery disease [8, 10, 20, 22, 24, 29, 31–34]. Besides, one of the studies excluded patients without previous echocardiography records [35]. Five studies carried out echocardiography due to clinical needs [18, 25, 26, 30, 32], while the protocols arranged the others. The timing of echocardiography was various in the enrolled studies with 13 studies within 24 h after admission [18–22, 25, 27–29, 31, 36–38], five within 48 h [8, 10, 23, 34, 35], and three within 72 h [26, 30, 32]. Two studies did not mention the time points [24, 33].

### Quality of enrolled studies

The detailed study appraisal in the qualitative synthesis is in Additional file 1: Appendix Table S2. Regarding the domain of selection, four cohort studies had partial representativeness because of the selection criteria and the indication of echocardiography [19, 29, 33, 35], and two did not have a secure record regarding ascertainment of exposure due to lacking detailed echocardiography operation times [34, 36]. The studies’ risk estimates are listed in Additional file 1: Appendix Table S3 and Figure S1. Among them, 14 studies provided risk estimate adjustment in the comparison. (Additional file 1: Appendix Table S6). All studies confirmed the outcomes by medical records, and phone calls were used to verify the survival status if the patients were discharged before the study period. Follow-up duration varied according to the study designs. The two case–control studies had the same method of ascertainment for cases and controls, and the selection and definition of controls were clear. Among them, only one study conducted further analysis to improve the comparability [24].

### Outcomes

**Fig. 2:**
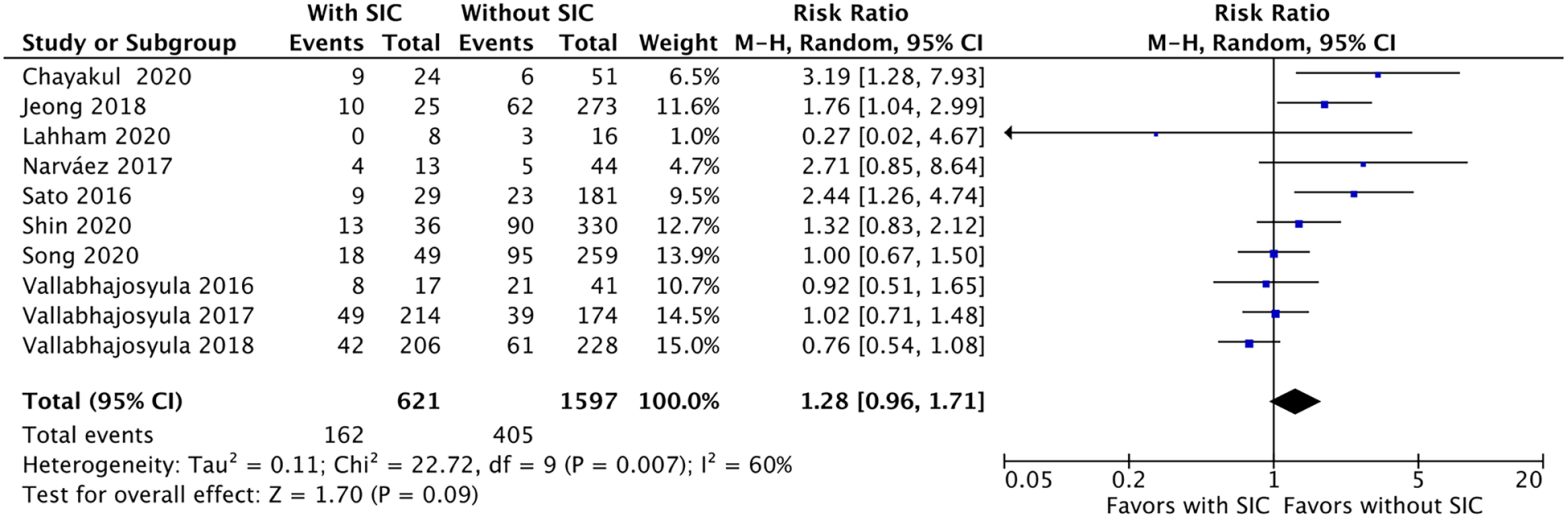
The forest plot shows in-hospital mortality between septic patients with and without sepsis-induced cardiomyopathy (SIC). SIC is non-statistically associated with higher risk of in-hospital mortality among septic patients

Figure 2 presents the RR of in-hospital mortality in patients with and without SIC. Ten articles were pooled [25–30, 33–35, 38], while in-hospital mortality was 26.0% (162/621) in the SIC group and 25.3% (405/1597) in the non-SIC group. Compared to the non-SIC group, SIC was non-statistically associated with an increased risk of in-hospital mortality (RR 1.28, [95% CI 0.96 to 1.71]; *p* = 0.09, *I*^2^ = 60%).

**Fig. 3:**
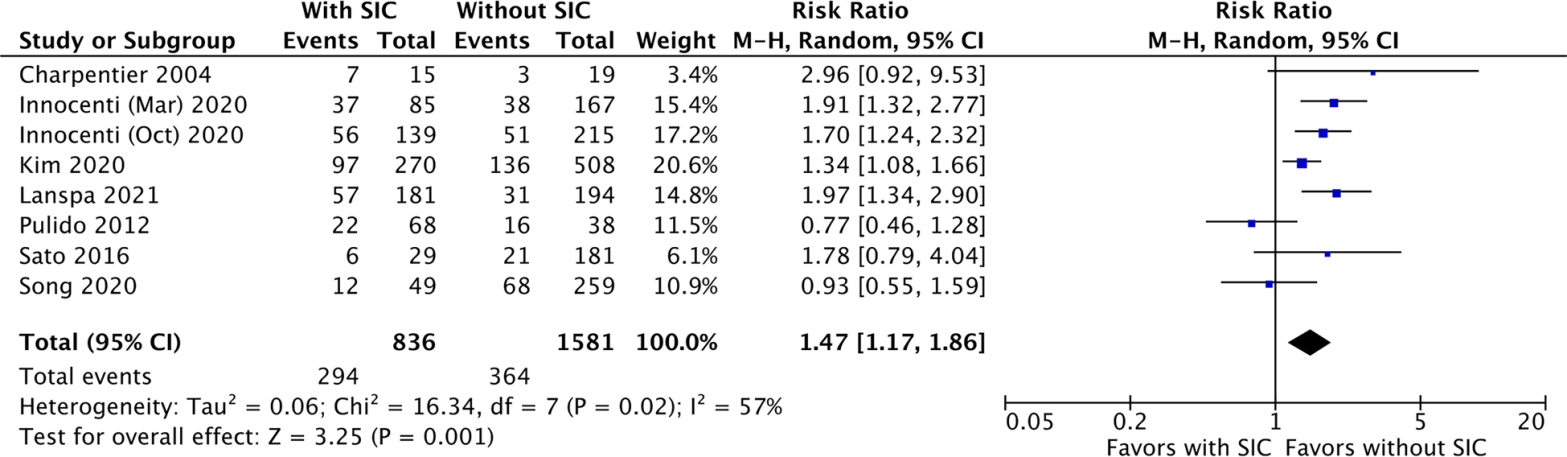
The forest plot shows one-month mortality between septic patients with and without sepsis-induced cardiomyopathy (SIC). SIC is significantly related to higher risk of one-month mortality among septic patients

Eight studies were included to compare 1-month mortality between septic patients with and without SIC [10, 18, 22, 25, 32, 35–37]. Fig. 3 presents the incidence of both groups and the RR of 1-month mortality. The 1-month mortality rate was 35.2% (294/836) and 23.0% (364/1581) for patients with and without SIC. Patients with SIC were significantly associated with a higher risk of 1-month mortality than those without SIC (RR 1.47, [95% CI 1.17 to 1.86]; *p* < 0.01, *I*^2^ = 57%).

### Subgroup and sensitivity analyses

**Fig. 4:**
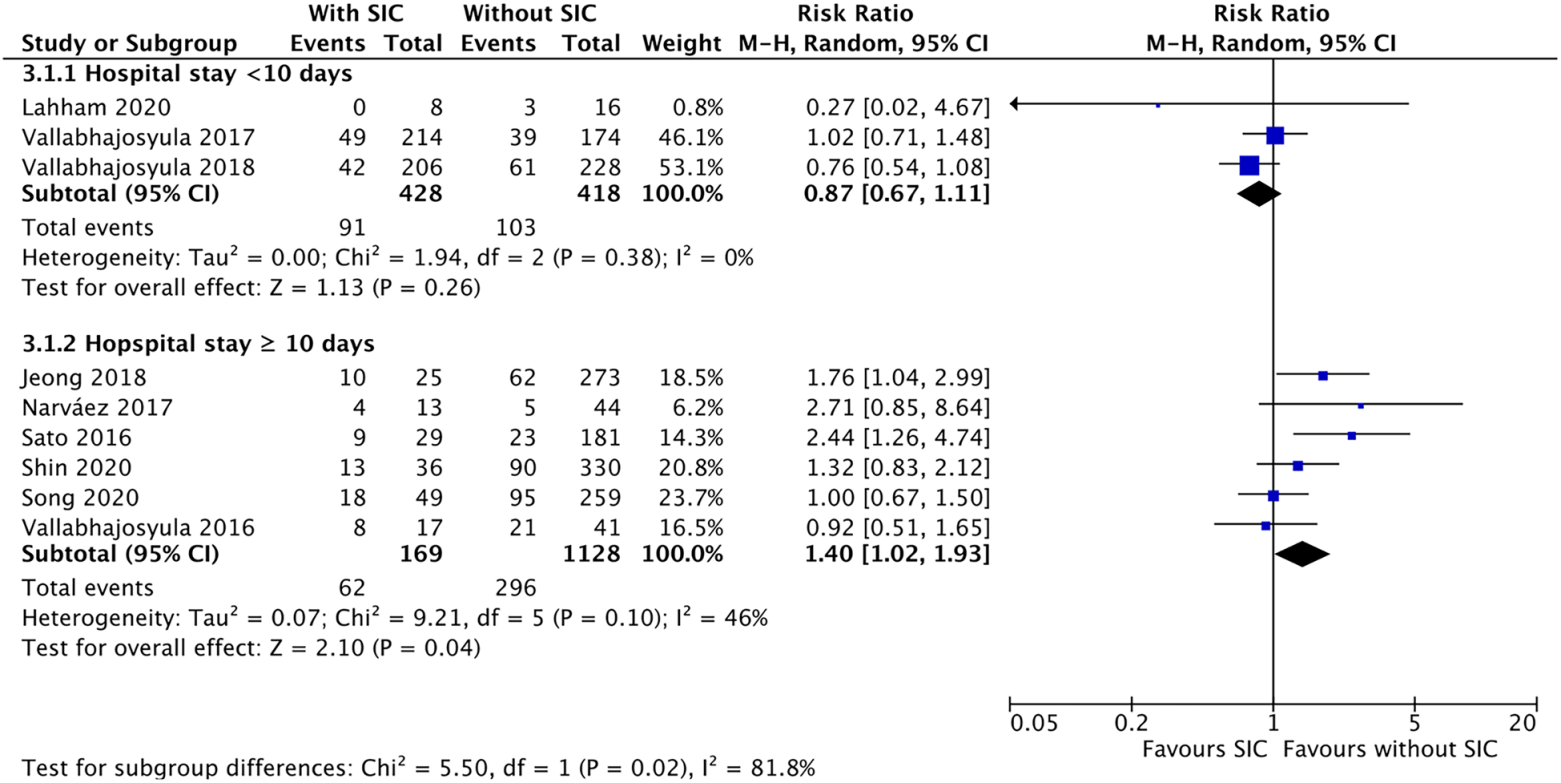
The forest plot presents the result of a subgroup analysis dividing in-hospital mortality into shorter (< 10 days, upper part) and longer (≥ 10 days, lower part) lengths of hospital stay. SIC is associated with a higher risk of in-hospital mortality among septic patients with a length of hospital stay longer ≥ 10 days

Figure 4 presents the subgroup analysis of in-hospital mortality. For the group of hospital stay length  < 10 days, SIC was not associated with increased in-hospital mortality (RR 0.87, [95% CI 0.67 to 1.11]; *p* = 0.26, *I*^2^ = 0%); however, given the length  ≥ 10 days, SIC was significantly associated with a higher risk of the mortality, compared to those without SIC (RR 1.40, [95% CI 1.02 to 1.93]; *p* = 0.04, *I*^2^ = 46%). The heterogeneity of in-hospital mortality decreased in the subgroups, i.e., the *I*^2^ value decreased from 60 to 0% and 46%, respectively.

**Fig. 5:**
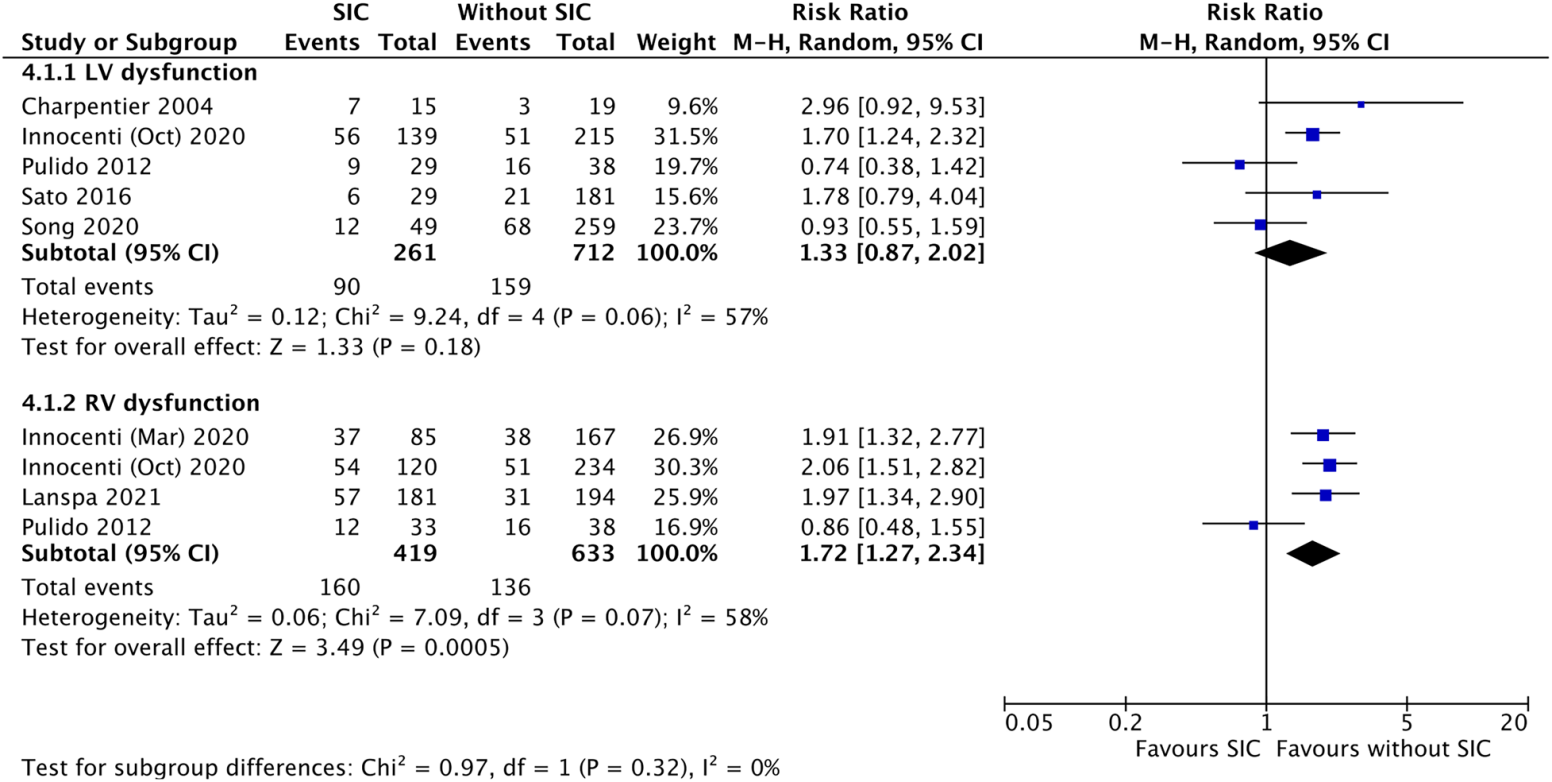
The forest plot presents the subgroup-analysis result stratifying one-month mortality by left ventricular (LV) and right ventricular (RV) dysfunction. Sepsis-induced cardiomyopathy (SIC) with RV dysfunction is significantly associated with higher risk of one-month mortality (lower part), while the association between SIC with LV dysfunction and one-month mortality is insignificant (upper part)

Figure 5 presents the subgroup analysis to examine the impact of LV and RV dysfunction on 1-month mortality. SIC with RV dysfunction was significantly related to an increased risk of 1-month mortality among patients with SIC, compared to those without SIC (RR 1.72, [95% CI 1.27 to 2.34]; *p* < 0.01, *I*^2^ = 58%), while LV dysfunction was not (RR 1.33, [95% CI 0.87 to 2.02]; *p* = 0.18, *I*^2^ = 57%).

The results of other subgroup analyses are shown in Additional file 1: Appendix Figures S5–S11 and S16–S22. The between-subgroup heterogeneity in each analysis was insignificant, except for the echocardiography operation timing in the in-hospital mortality (*p* = 0.02). The RRs on Days 1, 2, and 3 were 1.86 (1.18–2.93, *p* = 0.01), 1.13 (0.83–1.53, *p* = 0.44), and 0.88 (0.66–1.17, *p* = 0.08), respectively. (Additional file 1: Appendix Figure S6) The meta-regression results are summarized in Additional file 1: Appendix Tables S7 and S8 and Additional file 1: Appendix Figures S12 and S23. The results showed the factor’s effect direction (positive for age; negative for echocardiography operation timing, SOFA scores, and the percentages of mechanical ventilation and septic shock) and the heterogeneity from which studies, but the influences were insignificant in the outcome heterogeneity.

A one-by-one exclusion method was used for sensitivity analysis, and the results showed that the effect of an individual article on risk ratio and heterogeneity was small. (Additional file 1: Appendix Tables S5 and S6) The other sensitivity analyses did not change the original findings of in-hospital (RRs range between 1.31–1.39) and 1-month mortality (RRs range 1.40–1.52) when taking into account potential duplicate patients and uncertain extracted data. (Additional file 1: Appendix Figures S2–S4 and S13–S15).

## Discussion

### Clinical implication

In this systematic review and meta-analysis of observational studies, our findings show that despite a marginal increase, the in-hospital mortality of SIC patients was non-statistically higher than that of non-SIC patients, consistent with previous findings [39]. However, the length of hospital stay ranged substantially across the enrolled studies (4.9–43 days), and this heterogeneity may confound the result. In our analyses, SIC was associated with higher risks of 1-month mortality among septic patients even after discharge; moreover, given the hospital stay length  ≥ 10 days, SIC patients may have a 1.4-time risk of in-hospital mortality, compared with non-SIC patients. The findings are contrary to some studies that indicated septic patients developing LVSD might even have better survival than those with normal LV systolic function during sepsis [4, 9]. The discrepancy could be due to the fact that in the previous studies, the LVEF was influenced not only by cardiac contractility but also by preload and afterload. Therefore, the complex hemodynamic profiles may not have been fully quantified and accounted for in most critically ill septic patients [40]. Besides, the results of the previous studies could be biased due to the lack of adjustment of sepsis severity scores, different types of vasopressors, and other variables [2, 41].

SIC is usually considered a temporary and reversible phenomenon that mainly affects septic patients at the acute stage [4, 6]. The mechanism is still poorly understood. Some studies found that the associated mediators may include endotoxin, inflammatory cytokines (such as tumor necrosis factor α and interleukin-1), nitric oxide, mitochondrial dysfunction, downregulation of β-adrenergic receptors, and decreasing myofibril response to calcium and histone release [6, 42]. However, these mediators were mainly observed at the acute sepsis stage, and the duration of the impact of these chemical mediators and reactions on the myocardium is still arbitrary. Leah B. Kosyakovsky LB et al. indicated that people who survived sepsis might have long-term, higher risks of late cardiovascular events (1.77 and 1.67 times the risk of myocardial infarction and stroke, respectively) at least 5 years following hospital discharge [43]. A series of host responses to sepsis, like dysregulated inflammation, immune function, metabolism, endothelial dysfunction, and coagulation, are also causal mediators of cardiovascular diseases, e.g., atherosclerosis, thrombosis, and myocardial injury [44–46]. These responses may lead to long-term cardiovascular diseases rather than only short-term transient cardiovascular damage [47]. This implies that SIC-related responses may continue influencing the cardiovascular system or other systems even though the septic patients have recovered from cardiomyopathy [48]. In our findings, the mortality risk between SIC and non-SIC among septic patients with shorter hospital stay length (< 10 days) was not significantly different. However, SIC was associated with higher 1-month mortality among septic patients and increased in-hospital mortality for those with the length of hospital stay  ≥ 10 days. This phenomenon may imply that SIC patients potentially have more extended host responses to sepsis, contributing to higher mortality after the early acute stage. Also, these findings may emphasize the importance of continuous care and monitoring among SIC patients even after recovery from cardiomyopathy.

### Right and left ventricular dysfunction in sepsis

Sepsis and septic shock can depress myocardial function and lead to biventricular dysfunction. Our study found that LV dysfunction was not associated with SIC-related 1-month mortality. In contrast, RV dysfunction was associated with a higher risk of 1-month mortality. Our study did not categorize LV dysfunction into LVSD and LVDD because the articles meeting the inclusion criteria for acute-stage analysis were inadequate. However, LVDD is more likely to be associated with overall mortality than LVSD. A meta-analysis including 585 patients with poor LVEF presented that LVSD was neither a sensitive (52%) nor specific (63%) predictor of mortality among septic patients [11]. Similarly, another study pooling 636 patients demonstrated that LVSD was not associated with mortality; nevertheless, LVDD was associated with a 1.82-time risk of overall mortality [12]. Due to the controversial findings, more studies regarding SIC-related LV dysfunction may be warranted to examine the impact of LVSD and LVDD on mortality at the acute stage (in-hospital and 1-month mortality) among septic patients.

In past decades, right heart catheterization and radionuclide ventriculography have been used to evaluate RV dysfunction in septic patients [49]. More recent studies have used echocardiography to assess RV performance with advancements and convenience. This study included various evaluation tools and found that SIC patients with RV dysfunction have a 1.74-time risk of 1-month mortality. Preload failure, afterload-induced dysfunction, and ARDS all can lead to RV dysfunction, and studies have indicated RV dysfunction as an independent risk factor for in-hospital mortality among septic patients [3, 26, 50]. Besides, SIC patients with RV dysfunction may have lower cardiac output and use more inotropic medications, leading to more comorbidities, such as acute kidney injury, and, thus, higher mortality [26]. As demonstrated by Vallabhajosyula, S. et al., RV dysfunction is associated with short-term (≤ 30 days) and long-term [1–12 months] sepsis-related mortality [13]. With relatively lower heterogeneity, our study may provide further information to support the association of RV dysfunction and 1-month mortality among SIC patients. Also, the findings emphasize the importance of routine RV assessment for septic patients [13].

### Limitation

There are some limitations in this study. First, this meta-analysis pooled the data from various observational studies with different cut-off values, selection definitions, and severities of sepsis, which may lead to heterogenicity in the outcomes. Despite a large number of subgroup analyses and meta-regression, it was impossible to examine some sources of heterogeneity without individual-level patient data. For example, some pre-existing comorbidities, such as ischemic heart disorders, heart failure, or ARDS, that should have been excluded or could be the SIC risk factors or exacerbating variables, were not sufficiently explored. Second, unadjusted confounding is frequently a concern in observational studies. We directly extracted the original event numbers from the included studies to calculate risk estimates, and these estimates were not adjusted for confounding factors. In this case, the DerSimonian and Laird random-effects model minimized the impact. We then performed meta-regression and subgroup analyses to adjust the confounding and assess the heterogeneity. Despite this, the residue and unmeasurable effects could impact our results. Some outcomes with low significance or high heterogeneity may need a cautious interpretation. At last, this study conducted a systematic review of relevant studies and pooled data to investigate the association between SIC and mortality. The causal relationship may require future cohorts and biomechanical studies in the absence of detailed time sequences and other background determinants.

## Conclusions

Our study found that SIC is related to higher 1-month mortality among septic patients and increased in-hospital mortality among those with a more extended hospital stay than 10 days. Besides, RV dysfunction was found to be associated with a higher risk of 1-month mortality. These findings emphasize the importance of prompt biventricular assessment for septic patients, with continuum care and monitoring for SIC patients even after recovery. Future studies with a more explicit definition of sepsis, hemodynamic parameters, variables of assessment tools, and outcomes are warranted to provide more evidence of the association between SIC and mortality.

## Supplementary Information

**Additional file 1: Appendix Table S1. **Search strategy in PubMed and Embase on 8 July 2021. **Appendix Table S2. **Appraisal of cohort studies and case-control studies with Newcastle Ottawa Scale. **Appendix Table S3.** Risk estimates of the included studies. **Appendix Table S4.** Risk adjustment method of the included studies for data pooling. **Appendix Table S5. **One-by-one exclusion method for subgroup analysis of in-hospital mortality. **Appendix Table S6. **One-by-one exclusion method for sensitivity analysis of one-month mortality. **Appendix Table S7.** A random-effects meta-regress with Egger's regression-based test for the in-hospital mortality. **Appendix Table S8.** A random-effects meta-regress with Egger's regression-based test for the one-month mortality. **Appendix Figure S1.** The forest plot of the total selected studies 1. Mortality during ICU stay; 2. Mortality within 7 days; 3. Mortality within 10 days; 4. One-month mortality; 5. In-hospital morality; 6. One-year mortality; 7. Two-year mortality; 8. Mortality with non-defined duration. **Appendix Figure S2.** Sensitivity analyses of in-hospital mortality. Using the data for left ventricular diastolic dysfunction in Vallabhajosyula et al. 2016. **Appendix Figure S3.** Sensitivity analyses of in-hospital mortality. Using the data for left ventricular diastolic and diastolic dysfunction in Vallabhajosyula et al. 2016, with the assumption of no duplicated patients. **Appendix Figure S4. **Sensitivity analysis for in-hospital mortality. Due to the possibility of duplicated patients, the sensitivity analysis excluded anyone study of Vallabhajosyula to evaluate the range of result uncertainty. (upper, excluding Vallabhajosyula, 2017; lower, excluding Vallabhajosyula, 2018). **Appendix Figure S5. **Subgroup analysis for in-hospital mortality. The selected studies were divided into sepsis diagnosis with sepsis II and sepsis III definitions. (1, sepsis II; 2, sepsis III). **Appendix Figure S6. **Subgroup analysis for in-hospital mortality. The selected studies were divided into Day-1, Day-2, Day-3 echocardiography screening. (1, Day 1; 2, Day 2; 3, Day 3). **Appendix Figure S7. **Subgroup analysis for in-hospital mortality. The selected studies were divided into echocardiography by protocol and by clinical needs. (1, by protocol; 2, by clinical needs). **Appendix Figure S8. **Subgroup analysis for in-hospital mortality. The selected studies were divided into the subgroups according to (1) left ventricular systolic dysfunction, (2) left ventricular diastolic dysfunction, (3) left ventricular dysfunction, (4) right ventricular dysfunction. **Appendix Figure S9. **Subgroup analysis for in-hospital mortality. The selected studies were divided into the subgroups according to different cut-off values. 1. Only LVEF <50%, 2. LVEF<50%, or LVEF reduction >10%, 3. LVEF<50%+E/e’>15, 4. RV S’ <15cm/s or TAPSE <16mm. **Appendix Figure S10. **Subgroup analysis for in-hospital mortality. The selected studies were divided into the subgroups according to whether the risk estimate adjustment was performed in the selected studies. (1, without risk estimate adjustment; 2, with risk estimate adjustment). **Appendix Figure S11. **Subgroup analysis for in-hospital mortality. The selected studies were divided into the subgroups according to the appraisal quality of studies (1. Worse quality; 2. Better quality). **Appendix Figure S12. **The bubble plot diagrams showed the meta-regression examining the heterogeneity in in-hospital mortality by different characteristics in the selected studies. **Appendix Figure S13. **Sensitivity analyses of one-month mortality. Using the data for right ventricular dysfunction in Innocenti’s study. **Appendix Figure S14. **Sensitivity analyses of one-month mortality. Using the data for left and right ventricular dysfunction in Innocenti’s study Innocenti’s data (assumed no duplicated patients). **Appendix Figure S15. **Sensitivity analysis for one-month mortality. Due to the possibility of duplicated patients, the sensitivity analysis excluded anyone study of Innocenti to evaluate the range of result uncertainty. (upper, excluding Innocenti, Mar 2020; lower, excluding Innocenti, Oct 2020). **Appendix Figure S16. **Subgroup analysis for one-month mortality. The selected studies were divided into sepsis diagnosis with sepsis II and sepsis III definitions. (1, sepsis II; 2, sepsis III). **Appendix Figure S17. **Subgroup analysis for one-month mortality. The selected studies were divided into Day-1, Day-2, Day-3 echocardiography screening. (1, Day 1; 2, Day 2; 3, Day 3). **Appendix Figure S18. **Subgroup analysis for one-month mortality. The selected studies were divided into echocardiography by protocol and by clinical needs. (1, by protocol; 2, by clinical needs). **Appendix Figure S19. **Subgroup analysis for one-month mortality. The selected studies were divided into the subgroups according to (1) left ventricular systolic dysfunction, (2) left ventricular diastolic dysfunction, (3) left ventricular dysfunction, (4) right ventricular dysfunction, (5) left and right ventricular dysfunction. **Appendix Figure S20. **Subgroup analysis for one-month mortality. The selected studies were divided into the subgroups according to different cut-off values. 1. Only LVEF <50%, 2. LVEF<50% or LVEF reduction >10%, 3. LVEF<50%+E/e’>15, 4. RV S’ <15cm/s or TAPSE <16mm. **Appendix Figure S21. **Subgroup analysis for one-month mortality. The selected studies were divided into the subgroups according to the appraisal quality of studies (1. Worse quality; 2. Better quality). **Appendix Figure S22. **Subgroup analysis for one-month mortality. The selected studies were divided into the subgroups according to whether the risk estimate adjustment was performed in the selected studies. (1, without risk estimate adjustment; 2, with risk estimate adjustment). **Appendix Figure S23. **The bubble plot diagrams showed the meta-regression examining the heterogeneity in one-month mortality by different characteristics in the selected studies.

## Data Availability

All data generated or analyzed during this study are included in this published article and its supplementary information files.

## Abbreviations

SIC: Sepsis-induced cardiomyopathy
LV: Left ventricular
LVSD: Left ventricular systolic dysfunction
LVDD: Left ventricular diastolic dysfunction
RV: Right ventricular
APACHE score: Acute physiology and chronic health evaluation score
SOFA score: Sequential organ failure assessment score
PRISMA guidelines: Preferred reporting items for systemic reviews and meta-analyses guidelines
RR: Risk ratio
CI: Confidence interval
LVEF: Left ventricular ejection fraction
ARDS: Acute respiratory distress syndrome
