# Supplementary material for: Association of sepsis-induced cardiomyopathy and mortality: a systematic review and meta-analysis
Source: Ann Intensive Care. 2022 Dec 13;12:112. doi: 10.1186/s13613-022-01089-3 (PMC9748009; doi:10.1186/s13613-022-01089-3)

**Association of Sepsis-induced Cardiomyopathy and Mortality:**

**A systematic review and meta-analysis**

Yu-Min Lin MD^1^, Mei-Chuan Lee MSc^2,3^, Han Siong Toh MD^4,5,6^, Wei-Ting Chang MD^1,5,7^, Sih-Yao Chen MD^1^, Fang-Hsiu Kuo MSc^1^, Hsin-Ju Tang PhD^8^, Yi-Ming Hua BS^2^, Dongmei Wei MD^9^, Jesus Melgarejo MD^9^, Zhen-Yu Zhang MD, PhD^9^, Chia-Te Liao MD^1,3,9^

**Additional file**

1. **Appendix tables**

**Appendix Table 1.** Search strategy in PubMed and Embase on 8 July 2021. Page 4

**Appendix Table 2.** Appraisal of cohort studies and case-control studies with Newcastle Ottawa Scale. Page 5-6

**Appendix Table 3.** Risk estimates of the included studies. Page 7

**Appendix Table 4.** Risk adjustment method of the included studies for data pooling. Page 8-9

**Appendix Table 5.** One-by-one exclusion method for subgroup analysis of in-hospital mortality Page 10

**Appendix Table 6.** One-by-one exclusion method for sensitivity analysis of one-month mortality Page 11

**Appendix Table 7.** A random-effects meta-regress with Egger's regression-based test for the in-hospital mortality. Page 12

**Appendix Table 8.** A random-effects meta-regress with Egger's regression-based test for the one-month. mortality. Page 13

1. **Appendix figures**

**Appendix Figure 1.** The forest plot of the total selected studies 1. Mortality during ICU stay; 2. mortality within 7 days; 3. Mortality within 10 days; 4. One-month mortality; 5. In-hospital morality; 6. One-year mortality; 7. Two-year mortality; 8. mortality with non-defined duration. Page 14

*In-hospital mortality*

**Appendix Figure 2.** Sensitivity analyses of in-hospital mortality. Using the data for left ventricular diastolic dysfunction in Vallabhajosyula et al. 2016. Page 15

**Appendix Figure 3.** Sensitivity analyses of in-hospital mortality. Using the data for left ventricular diastolic and diastolic dysfunction in Vallabhajosyula et al. 2016, with the assumption of no duplicated patients. Page 16

**Appendix Figure 4.** Sensitivity analysis for in-hospital mortality. Due to the possibility of duplicated patients, the sensitivity analysis excluded anyone study of Vallabhajosyula to evaluate the range of result uncertainty. (upper, excluding Vallabhajosyula, 2017; lower, excluding Vallabhajosyula, 2018). Page 17

**Appendix Figure 5.** Subgroup analysis for in-hospital mortality. The selected studies were divided into sepsis diagnosis with sepsis II and sepsis III definitions. (1, sepsis II; 2, sepsis III). Page 18

**Appendix Figure 6.** Subgroup analysis for in-hospital mortality. The selected studies were divided into Day-1, Day-2, Day-3 echocardiography screening. (1, Day 1; 2, Day 2; 3, Day 3). Page 19

**Appendix Figure 7.** Subgroup analysis for in-hospital mortality. The selected studies were divided into echocardiography by protocol and by clinical needs. (1, by protocol; 2, by clinical needs). Page 20

**Appendix Figure 8.** Subgroup analysis for in-hospital mortality. The selected studies were divided into the subgroups according to (1) left ventricular systolic dysfunction, (2) left ventricular diastolic dysfunction, (3) left ventricular dysfunction, (4) right ventricular dysfunction. Page 21

**Appendix Figure 9.** Subgroup analysis for in-hospital mortality. The selected studies were divided into the subgroups according to different cut-off values. 1. Only LVEF <50%, 2. LVEF<50% or LVEF reduction >10%, 3. LVEF<50%+E/e’>15, 4. RV S’ <15cm/s or TAPSE <16mm. Page 22

**Appendix Figure 10.** Subgroup analysis for in-hospital mortality. The selected studies were divided into the subgroups according to whether the risk estimate adjustment was performed in the selected studies. (1, without risk estimate adjustment; 2, with risk estimate adjustment). Page 23

**Appendix Figure 11.** Subgroup analysis for in-hospital mortality. The selected studies were divided into the subgroups according to the appraisal quality of studies (1. Worse quality; 2. Better quality). Page 24

**Appendix Figure 12.** The bubble plot diagrams showed the meta-regression examining the heterogeneity in in-hospital mortality by different characteristics in the selected studies. Page 25

*One-month mortality*

**Appendix Figure 13.** Sensitivity analyses of one-month mortality. Using the data for right ventricular dysfunction in Innocenti’s study. Page 26

**Appendix Figure 14.** Sensitivity analyses of one-month mortality. Using the data for left and right ventricular dysfunction in Innocenti’s study Innocenti’s data (assumed no duplicated patients). Page 27

**Appendix Figure 15.** Sensitivity analysis for one-month mortality. Due to the possibility of duplicated patients, the sensitivity analysis excluded anyone study of Innocenti to evaluate the range of result uncertainty. (upper, excluding Innocenti, Mar 2020; lower, excluding Innocenti, Oct 2020). Page 28

**Appendix Figure 16.** Subgroup analysis for one-month mortality. The selected studies were divided into sepsis diagnosis with sepsis II and sepsis III definitions. (1, sepsis II; 2, sepsis III). Page 29

**Appendix Figure 17.** Subgroup analysis for one-month mortality. The selected studies were divided into Day-1, Day-2, Day-3 echocardiography screening. (1, Day 1; 2, Day 2; 3, Day 3). Page 30

**Appendix Figure 18.** Subgroup analysis for one-month mortality. The selected studies were divided into echocardiography by protocol and by clinical needs. (1, by protocol; 2, by clinical needs). Page 31

**Appendix Figure 19.** Subgroup analysis for one-month mortality. The selected studies were divided into the subgroups according to (1) left ventricular systolic dysfunction, (2) left ventricular diastolic dysfunction, (3) left ventricular dysfunction, (4) right ventricular dysfunction, (5) left and right ventricular dysfunction. Page 32

**Appendix Figure 20.** Subgroup analysis for one-month mortality. The selected studies were divided into the subgroups according to different cut-off values. 1. Only LVEF <50%, 2. LVEF<50% or LVEF reduction >10%, 3. LVEF<50%+E/e’>15, 4. RV S’ <15cm/s or TAPSE <16mm. Page 33

**Appendix Figure 21.** Subgroup analysis for one-month mortality. The selected studies were divided into the subgroups according to the appraisal quality of studies (1. Worse quality; 2. Better quality). Page 34

**Appendix Figure 22.** Subgroup analysis for one-month mortality. The selected studies were divided into the subgroups according to whether the risk estimate adjustment was performed in the selected studies. (1, without risk estimate adjustment; 2, with risk estimate adjustment). Page 35

**Appendix Figure 23.** The bubble plot diagrams showed the meta-regression examining the heterogeneity in one-month mortality by different characteristics in the selected studies. Page 36

| **Appendix Table 1.** Search strategy in PubMed and Embase on 8 July 2021 | | |
| --- | --- | --- |
| **PubMed database** | | |
| **No** | **Query** | **Results** |
| **#1** | “Sepsis”[Mesh] | 129,133 |
| **#2** | sepsis or septic | 219,138 |
| **#3** | #1 OR #2 | 219,138 |
| **#4** | ((((((“Ventricular Dysfunction”[Mesh]) OR “Ventricular Function”[Mesh]) OR “Myocardial Contraction”[Mesh]) OR “Heart Ventricles”[Mesh]) OR “Cardiomyopathies”[Mesh]) OR “Heart Failure”[Mesh]) OR “Heart Diseases”[Mesh] | 1,243,501 |
| **#5** | (Heart OR Cardia* OR Myocardi* OR Ventric*) AND (function* OR Contraction* OR failure OR decompensation OR incompetence OR insufficiency OR impairment OR myopath* OR disorder* OR depression) | 837,955 |
| **#6** | #4 OR #5 OR Cardiomyopath* OR Myocardiopath* | 1620,673 |
| **#7** | “Mortality”[Mesh] | 402,396 |
| **#8** | mortalit* OR death OR fatalit* | 2,010,128 |
| **#9** | #7 OR #8 | 2,106,605 |
| **#10** | #3 AND #6 AND #9 | 8,013 |
| **#11** | #10 AND (cohort studies[mh] OR cohort*[tw] OR controlled clinical trial[pt] OR case-control studies[mh] OR (case*[tw] AND (control*[tw] OR crossover[tw] OR cross-over[tw] OR comparison*[tw])) “control group”[tw] OR “control groups”[tw] OR risk*[tw] OR incidence*[tw] OR (epidemiologic methods[mh:noexp] AND (“1966”[dp]:”1989”[dp]))) | 3,589 |
| **Embase database** | | |
| **No** | **Query** | **Results** |
| **#1** | 'sepsis'/exp/mj | 110,552 |
| **#2** | sepsis:ti,ab,kw OR septic:ti,ab,kw | 217,619 |
| **#3** | #1 OR #2 | 259,523 |
| **#4** | 'cardiomyopathy'/exp/mj OR  'heart ventricle function'/exp/mj OR  'heart contraction'/exp/mj OR  'heart failure'/exp/mj OR  'heart stress'/exp/mj OR  'heart movement'/exp/mj OR  'heart performance'/exp/mj OR | 375,532 |
| **#5** | (heart OR cardia* OR myocardi* OR ventric*) NEAR/3 (function* OR contraction* OR failure OR decompensation OR incompetence OR insufficiency OR impairment OR myopath* OR disorder* OR depression) | 703,593 |
| **#6** | #4 OR #5 OR Cardiomyopath* OR Myocardiopath* | 867,708 |
| **#7** | 'mortality'/exp/mj | 174,998 |
| **#8** | mortalit*:ti,ab,kw OR death:ti,ab,kw OR fatalit*:ti,ab,kw | 2,128,184 |
| **#9** | #7 OR #8 | 2,150,788 |
| **#10** | #3 AND #6 AND #9 | 6,350 |
| **#11** | #10 AND ('cohort analysis'/exp OR 'longitudinal study'/exp OR 'prospective study'/exp OR 'follow up'/exp OR cohort* OR 'case control study'/exp OR (case* NEXT/5 (control* OR crosscover OR cross-over)) OR (case NEXT/3 comparision*) OR "control group*" OR risk* OR incidence*) | 4,000 |

| **Appendix Table 2.** Appraisal of cohort studies and case-control studies with Newcastle Ottawa Scale | | | | | | | | | |
| --- | --- | --- | --- | --- | --- | --- | --- | --- | --- |
| **Cohort study** | | | | | | | | | |
| **Study** | **Selection** | | | | **Comparability** | **Outcome** | | | **Total score** |
|  | **Representativeness of the exposed cohort** | **Selection of non-exposed cohort** | **Ascertainment of exposure** | **Demonstration that outcome of interest was not present at start of the study** | **Comparability of cohorts on the basis of the design or analysis** | **Assessment of outcome** | **Was follow-up long enough for outcomes to occur** | **Adequacy of follow up of cohorts** |  |
| **Sato et al.**  **(Sep.2016)** | ★ | ★ | ★ | ★ |  | ★ | ★ | ★ | ★★★★★★★ |
| **Charpentier et al.**  **(Aug.2004)** | ★ | ★ | ★ | ★ |  | ★ | ★ | ★ | ★★★★★★★ |
| **Vallabhajosyula et al.**  **(Nov.2016)** | ★ | ★ | ★ | ★ |  | ★ | ★ | ★ | ★★★★★★★ |
| **Vallabhajosyula et al.**  **(Sep.2017)** |  | ★ | ★ | ★ |  | ★ | ★ | ★ | ★★★★★★ |
| **Boissier et al.**  **(May.2017)** | ★ | ★ | ★ | ★ |  | ★ | ★ |  | ★★★★★★ |
| **Vallabhajosyula et al.**  **(Feb.2018)** |  | ★ | ★ | ★ |  | ★ | ★ | ★ | ★★★★★★ |
| **Jeong et al.**  **(May.2018)** | ★ | ★ | ★ | ★ |  | ★ | ★ | ★ | ★★★★★★★ |
| **Rahasto et al.**  **(Jan.2019)** | ★ | ★ |  | ★ |  | ★ |  | ★ | ★★★★★ |
| **Lahham et al.**  **(Jan.2020)** | ★ | ★ |  | ★ |  | ★ | ★ | ★ | ★★★★★★ |
| **Innocenti et al.**  **(Oct.2020)** | ★ | ★ | ★ | ★ |  | ★ | ★ | ★ | ★★★★★★★ |
| **Shin et al.**  **(Mar.2020)** | ★ | ★ | ★ | ★ |  | ★ | ★ | ★ | ★★★★★★★ |
| **Chayakul et al.**  **(Dec.2020)** | ★ | ★ | ★ | ★ |  | ★ | ★ | ★ | ★★★★★★★ |
| **(Continued)** | | | | | | | | | |
| **Study** | **Selection** |  |  |  | **Comparability** | **Outcome** |  |  | **Total score** |
|  | **Representativeness of the exposed cohort** | **Selection of non-exposed cohort** | **Ascertainment of exposure** | **Demonstration that outcome of interest was not present at start of the study** | **Comparability of cohorts on the basis of the design or analysis** | **Assessment of outcome** | **Was follow-up long enough for outcomes to occur** | **Adequacy of follow up of cohorts** |  |
| **Narváez et al.**  **(Nov.2017)** | ★ | ★ | ★ | ★ |  | ★ | ★ | ★ | ★★★★★★★ |
| **Song et al.**  **(Apr.2020)** | ★ | ★ | ★ | ★ |  | ★ | ★ | ★ | ★★★★★★★ |
| **Innocenti et al.**  **(Mar.2020)** | ★ | ★ | ★ | ★ |  | ★ | ★ | ★ | ★★★★★★★ |
| **Kim et al.**  **(Jul.2020)** |  | ★ | ★ | ★ |  | ★ | ★ | ★ | ★★★★★★ |
| **Lanspa et al.**  **(Oct.2020)** |  | ★ | ★ | ★ |  | ★ | ★ | ★ | ★★★★★★ |
| **Pulido et al.**  **(Jun. 2012)** | ★ | ★ | ★ | ★ |  | ★ | ★ | ★ | ★★★★★★★ |
| **Artucio et al.**  **(Apr.1989)** | ★ | ★ | ★ | ★ |  | ★ |  | ★ | ★★★★★★ |
| **Landesberg1 et al.**  **(Apr.2012)** | ★ | ★ | ★ | ★ |  | ★ | ★ | ★ | ★★★★★★★ |
| **Wilhelm et al.**  **(Oct.2013)** | ★ | ★ | ★ | ★ |  | ★ | ★ | ★ | ★★★★★★★ |
| **Case-control study** | | | | | | | | | |
| **Study** | **Selection** |  |  |  | **Comparability** | **Outcome** |  |  | **Total score** |
|  | **Is the case definition adequate** | **Representativeness of the cases** | **Selection of Controls** | **Definition of Controls** | **Comparability of cases and controls on the basis of the design or analysis** | **Ascertainment of exposure** | **Same method of ascertainment for cases and controls** | **Non-Response rate** |  |
| **Prabhu et al.**  **(Mar.2015)** | ★ | ★ | ★ | ★ |  |  | ★ | ★ | ★★★★★★ |
| **Mourad et al.**  **(Sep.2013)** | ★ | ★ | ★ | ★ |  | ★ | ★ | ★ | ★★★★★★★ |
| Footnote: We appraised the representativeness of the exposed cohort based on the enrollment criteria in the individual studies. The study which enrolled the general and consecutive patients admitted to the emergency department or ICU due to sepsis and had small numbers of loss follow-up was scored as true representativeness of the average patients with sepsis-induced cardiomyopathy in the community. The four studies only had somewhat representativeness because Kim 2020 grouped the patients with normal cardiac troponin-I into the non-SIC, and the other three studies used echocardiography for diagnosis due to clinical needs, not by protocols. These may lead to the reduced representativeness of septic or SIC patients.  Regarding ascertainment of exposure, two studies were not scored as secure record because they lacked the clear timing of echocardiogram in their articles. | | | | | | | | | |

| **Appendix Table 3.** Risk estimates of the included studies | | | |
| --- | --- | --- | --- |
| **Study (year)** | In hospital mortality | One-month mortality | Other outcomes |
| **Artucio (1989)** | Risk ratio 0.53 |  |  |
| **Charpentier (2004)** |  | Risk ratio 2.96 |  |
| **Pulido (2012)** |  | Risk ratio 0.77 | One-year mortality  Risk ratio 1.89 |
| **Landesberg (2012)** | Hazard ratio: 2.9 |  |  |
| **Wilhelm (2013)** | Not applicable | | |
| **Mourad (2013)** |  |  | ICU mortality  Odds ratio 16.6 |
| **Prabhu (2015)** | Risk ratio: 3.71 |  |  |
| **Sato (2016)** | Risk ratio 2.44 | Risk ratio 1.78 |  |
| **Vallabhajosyula (2016)** | Risk ratio 0.92 |  |  |
| **Vallabhajosyula (2017)** | Risk ratio 1.02 |  | One-year mortality  Hazard ratio: 1.20 |
| **Narváez (2017)** | Risk ratio 2.71 |  | ICU mortality  Risk ratio: 3.38 |
| **Boissier (2017)** | Not applicable | | |
| **Vallabhajosyula (2018)** | Risk ratio 0.76 |  | Two-year mortality  Risk ratio: 0.93 |
| **Jeong (2018)** | Risk ratio 1.76 |  |  |
| **Rahasto (2019)** |  |  | 10-day mortality  Risk ratio: 1.60 |
| **Lahham (2020)** | Risk ratio 0.27 |  |  |
| **Shin (2020)** | Risk ratio 1.32 |  |  |
| **Innocenti (Mar.2020)** |  | Risk ratio 2.43 |  |
| **Song (2020)** | Risk ratio 1.00 | Risk ratio 0.93 | ICU mortality  Risk ratio 1.10 |
| **Kim (2020)** |  | Risk ratio 1.34 |  |
| **Lanspa (2020)** |  | Risk ratio 1.97 |  |
| **Innocenti (Oct.2020)** |  | Risk ratio 1.90 |  |
| **Chayakul (2020)** | Risk ratio 3.19 |  |  |

| **Appendix Table 4.** Risk adjustment method of the included studies for data pooling | |
| --- | --- |
| **Study (year)** | **Adjustment method** |
| **Artucio (1989)** | No risk adjustment for mortality |
| **Charpentier (2004)** | No risk adjustment for mortality |
| **Pulido (2012)** | No risk adjustment for mortality |
| **Landesberg (2012)** | Logistic regression analyses and multivariate regression analyses: basic characteristics and echocardiographic parameters |
| **Wilhelm (2013)** | Cox proportional analysis: APACHE-II score, low urine output, low LVSVI, lowest arterial oxygen saturation |
| **Mourad (2013)** | Multivariate logistic regression: SOFA score at admission, need for RRT, use of invasive MV |
| **Prabhu (2015)** | No risk adjustment for mortality |
| **Sato (2016)** | Multivariate logistic regression |
| **Vallabhajosyula (2016)** | No risk adjustment for mortality |
| **Vallabhajosyula (2017)** | Cox-proportional hazards model: Age, Charlson comorbidity index, APACHE III, septic shock, use of MV |
| **Narváez (2017)** | No risk adjustment for mortality |
| **Boissier (2017)** | No risk adjustment for mortality |
| **Vallabhajosyula (2018)** | No risk adjustment for mortality |
| **Jeong (2018)** | Multiple logistic regression analysis: age, sex, source of infection, underling comorbidities, heart rate, level of white blood count, erythrocyte sedimentation rate, C-reactive protein, procalcitonin, lactate, troponin, and NT pro-BNP, atrial fibrillation on admission, cardiomegaly, use of inotropic agents, and positive blood culture |
| **Rahasto (2019)** | Cox Regression model: ventricular function, cardiovascular biomarkers, venous oxygen saturation, creatinine level |
| **Lahham (2020)** | No risk adjustment for mortality |
| **Shin (2020)** | Cox regression analysis: Female gender, ICU stay, hospital stay, left atrial volume index |
| **Innocenti (Mar.2020)** | Cox proportional analysis: Age, gender, presence of shock, SOFA, NT-pro BNP and LVSD |
| **Song (2020)** | Cox proportional hazard regression analysis: APACHE II, delta neutrophil index, Lactate, LVEDD |
| **Kim (2020)** | Cox proportional |
| **Lanspa (2020)** | Multivariable logistic regression: Receipt of vasopressors, receipt of fluid, use of MV, APACHE II score |
| **Innocenti (Oct.2020)** | Cox proportional analysis: Age, septic shock, persistent high lactate levels, the presence of LVSD |
| **Chayakul (2020)** | Multivariable logistic regression: APACHE II score ≥20, HFpEF, use of MV, need for RRT |
| Abbreviation: APACHE=Acute Physiology and Chronic Health Evaluation, LVSVI=Left ventricular stroke volume index, SOFA=Sequential Organ Failure Assessment, RRT=Renal replacement therapy, MV=Mechanical ventilation, ICU=Intensive care unit, NT-proBNP=N terminal pro B type natriuretic peptide , LVSD=Left ventricular systolic dysfunction, LVEDD=Left ventricular end-diastolic diameter, HFpEF=Heart failure with preserved ejection fraction, ACP= afterload-related cardiac performance, CI=cardiac index, CPI=cardiac power index, PCT= procalcitonin, IL-6= interleukin 6 | |

| **Appendix Table 5.** One-by-one exclusion method for subgroup analysis of in-hospital mortality | | |
| --- | --- | --- |
| **Length of hospital stay < 10 days** | | |
| **Excluded study** | **Risk ratio (95% Confidence interval)** | ***I*^2^** |
| **None** | 0.87 (0.67, 1.11) | 0% |
| **Lahham 2020** | 0.88 (0.66, 1.17) | 22% |
| **Vallabhajosyula 2017** | 0.75 (0.53, 1.06) | 0% |
| **Vallabhajosyula 2018** | 1.00 (0.69, 1.44) | 0% |
| **Length of hospital stay ≥ 10 days** | | |
| **Excluded study** | **Risk ratio (95% Confidence interval)** | ***I*^2^** |
| **None** | 1.40 (1.02, 1.93) | 46% |
| **Jeong 2018** | 1.34 (0.93, 1.94) | 49% |
| **Narváez 2017** | 1.34 (0.97, 1.85) | 48% |
| **Sato 2016** | 1.26 (0.94, 1.69) | 29% |
| **Shin 2020** | 1.45 (0.97, 2.19) | 57% |
| **Song 2020** | 1.55 (1.09, 2.20) | 37% |
| **Vallabhajosyula 2016** | 1.53 (1.08, 2.16) | 46% |

| Appendix Table 6. One-by-one exclusion method for sensitivity analysis of one-month mortality | | |
| --- | --- | --- |
| Excluded study | **Risk ratio (95% Confidence interval)** | ***I*^2^** |
| None | 1.44 (1.08, 1.92) | 63% |
| Charpentier 2004 | 1.39 (1.04, 1.87) | 66% |
| Innocenti (Mar) 2020 | 1.37 (0.99, 1.89) | 63% |
| Innocenti (Oct) 2020 | 1.38 (1.00, 1.90) | 66% |
| Kim 2020 | 1.53 (1.10, 2.11) | 62% |
| Lanspa 2021 | 1.36 (0.99,1.87) | 62% |
| Pulido 2012 | 1.57 (1.20, 2.06) | 53% |
| Sato 2016 | 1.42 (1.04, 1.93) | 68% |
| Song 2020 | 1.53 (1.13, 2.08) | 64% |

| **Appendix Table 7.** A random-effects meta-regress with Egger's regression-based test for the in-hospital mortality | | | | | | |
| --- | --- | --- | --- | --- | --- | --- |
| Parameter | Coefficient | Standard error | t | Significant (2-tailed) | 95% Confidence interval, lower limit | 95% Confidence interval, upper limit |
| (Intercept) | -0.385 | 3.6087 | -0.107 | 0.925 | -15.912 | 15.142 |
| Standard error of effect size | 2.498 | 1.8846 | 1.325 | 0.316 | -5.611 | 10.607 |
| Screen day of echocardiogram | -0.112 | 0.1900 | -0.592 | 0.614 | -0.930 | 0.705 |
| Age | 0.014 | 0.0430 | 0.330 | 0.773 | -0.171 | 0.199 |
| SOFA Scores | -0.094 | 0.0618 | -1.521 | 0.268 | -0.360 | 0.172 |
| Mechanical ventilator (%) | -0.026 | 0.0363 | -0.708 | 0.608 | -0.487 | 0.436 |
| Septic shock (%) | -0.015 | 0.0214 | -0.704 | 0.609 | -0.256 | 0.286 |

| **Appendix Table 8.** A random-effects meta-regress with Egger's regression-based test for the one-month mortality | | | | | | |
| --- | --- | --- | --- | --- | --- | --- |
| Parameter | Coefficient | Standard error | t | Significant (2-tailed) | 95% Confidence interval, lower limit | 95% Confidence interval, upper limit |
| (Intercept) | -3.080 | 4.0959 | -0.752 | 0.590 | -55.123 | 48.963 |
| Standard error of effect size | -0.466 | 1.5464 | -0.301 | 0.814 | -20.115 | 19.183 |
| Screen day of echocardiogram | 0.100 | 0.1616 | 0.618 | 0.648 | -1.954 | 2.154 |
| Age | 0.059 | 0.0450 | 1.300 | 0.418 | -0.514 | 0.631 |
| SOFA Scores | -0.089 | 0.0964 | -0.918 | 0.527 | -1.314 | 1.137 |
| Mechanical ventilator (%) | -0.046 | 0.0256 | -1.809 | 0.322 | -0.372 | 0.279 |
| Septic shock (%) | -0.035 | 0.0224 | -1.555 | 0.364 | -0.250 | 0.319 |

**Appendix Figure 1.** The forest plot of the total selected studies 1. Mortality during ICU stay; 2. mortality within 7 days; 3. Mortality within 10 days; 4. One-month mortality; 5. In-hospital morality; 6. One-year mortality; 7. Two-year mortality; 8. mortality with non-defined duration


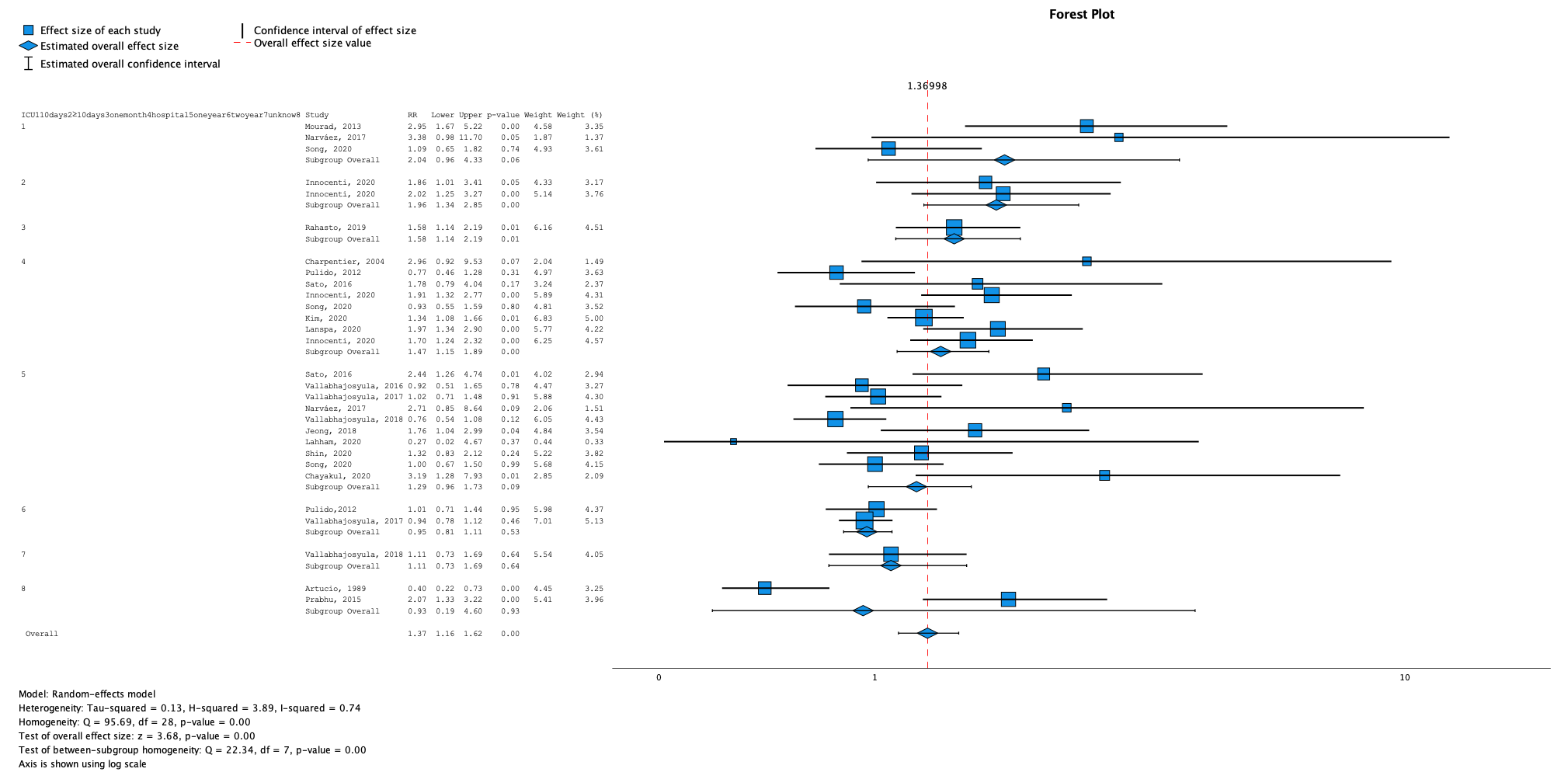


**Subgroup and sensitivity analyses for in-hospital mortality**

**Appendix Figure 2**. Sensitivity analyses of in-hospital mortality. Using the data for left ventricular diastolic dysfunction in Vallabhajosyula et al. 2016


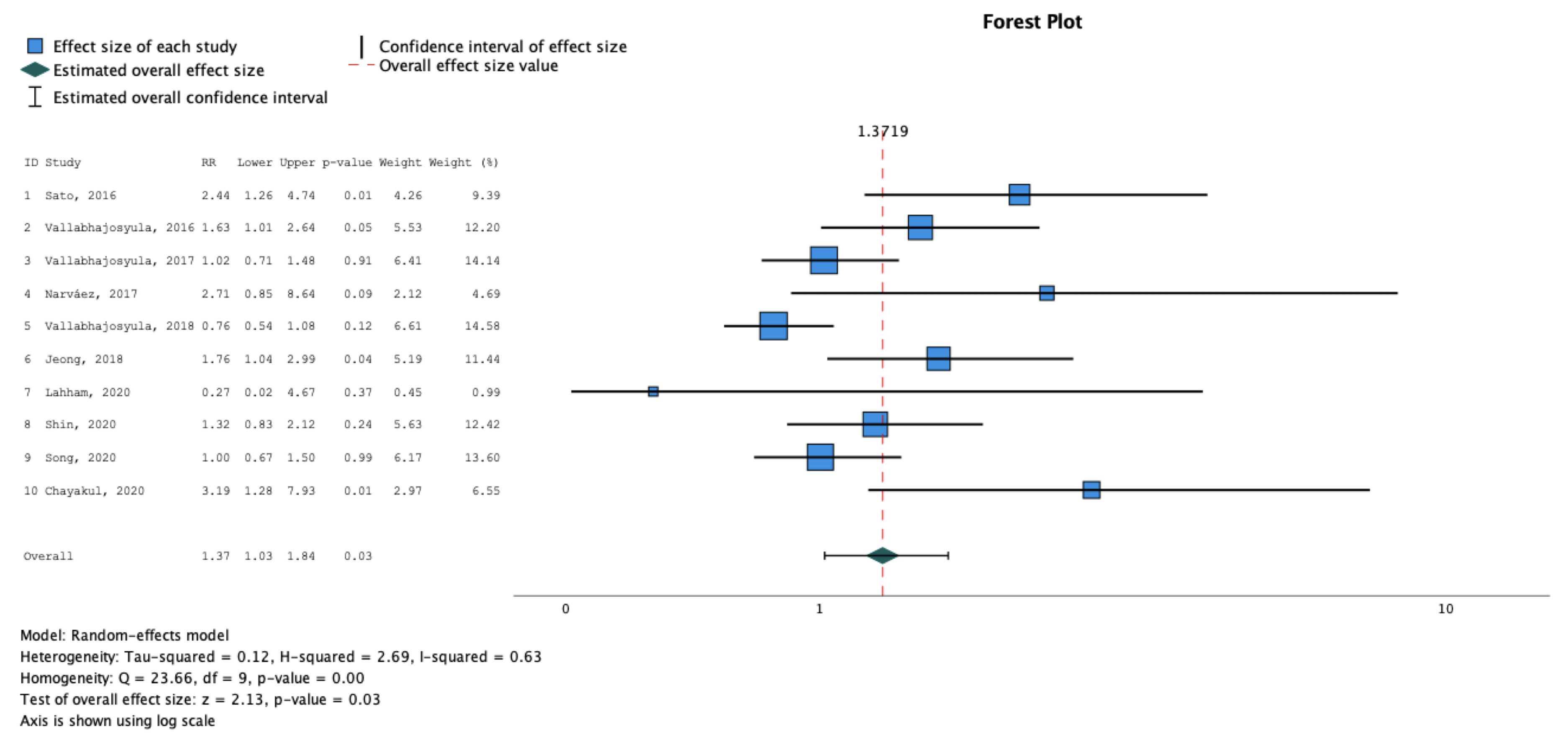


**Appendix Figure 3**. Sensitivity analyses of in-hospital mortality. Use the data for left ventricular systolic and diastolic dysfunction in Vallabhajosyula et al. 2016, with the assumption of no duplicated patients


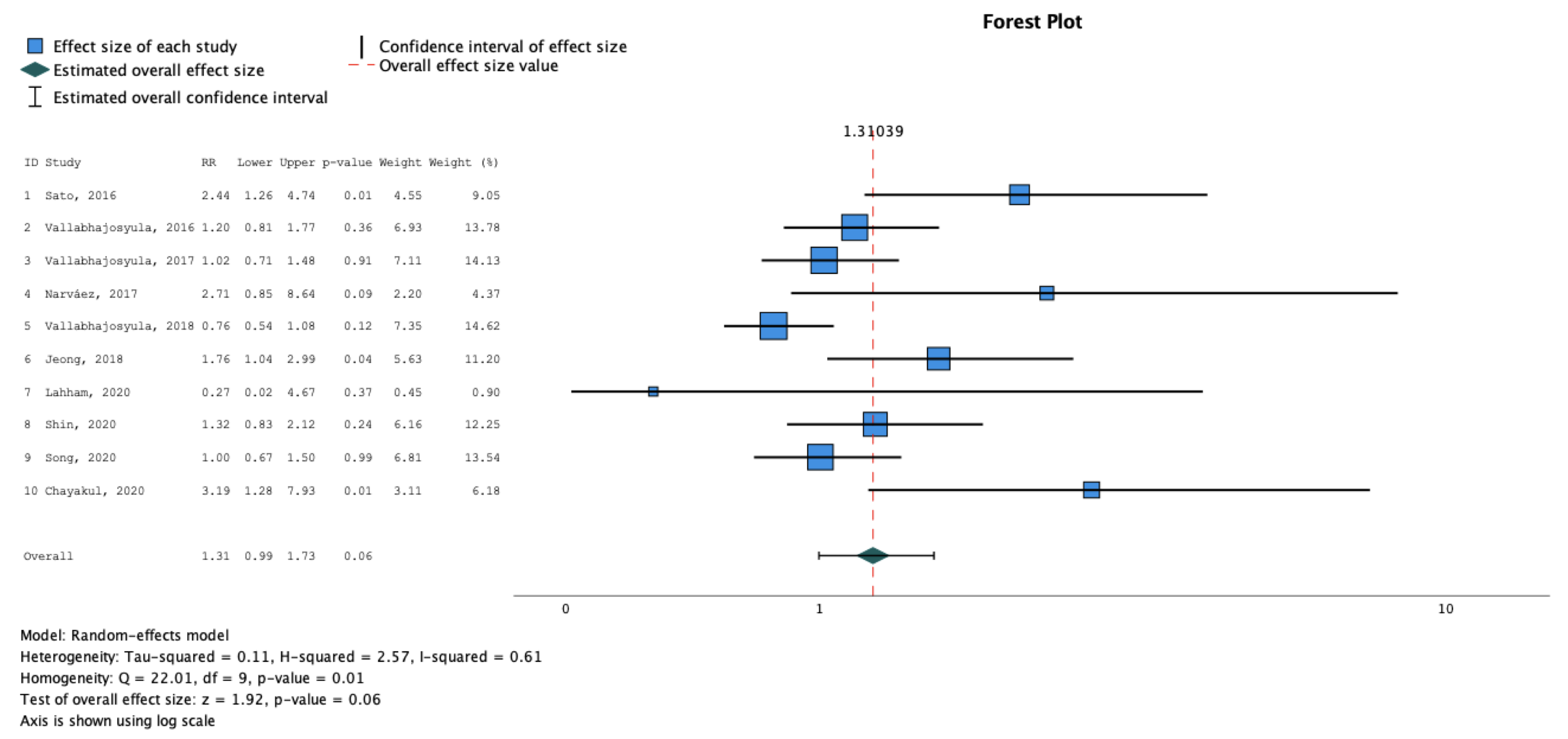


**Appendix Figure 4**. Sensitivity analysis for in-hospital mortality. Due to the possibility of duplicated patients, the sensitivity analysis excluded anyone study of Vallabhajosyula to evaluate the range of result uncertainty. (upper, excluding Vallabhajosyula, 2017; lower, excluding Vallabhajosyula, 2018)


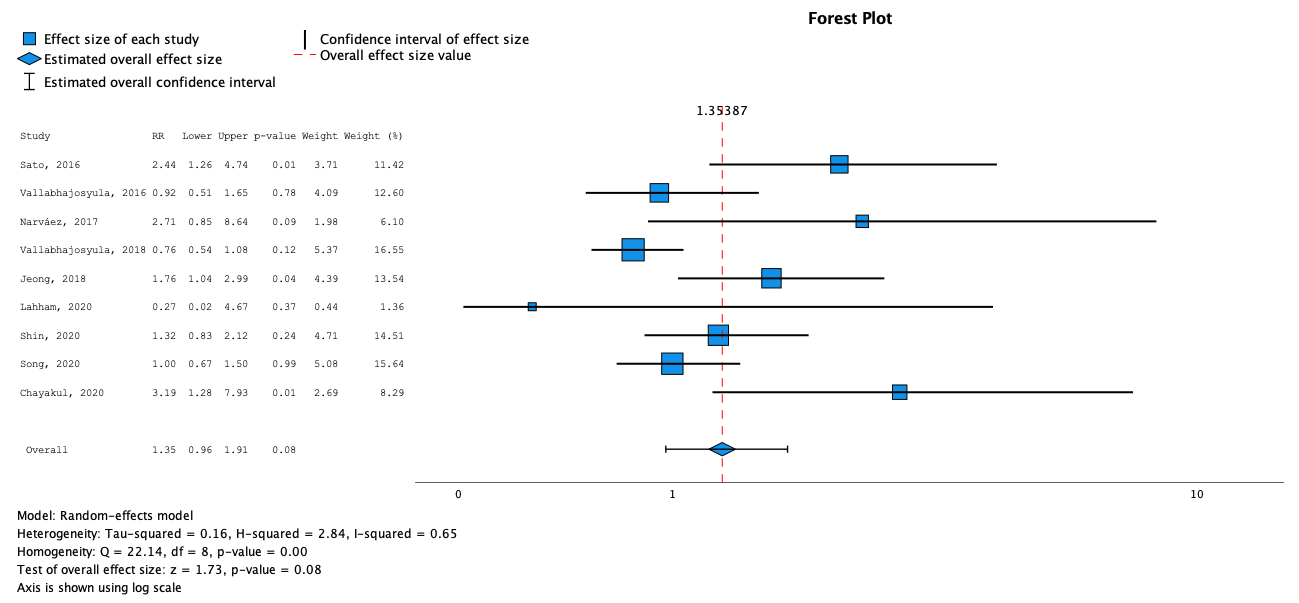


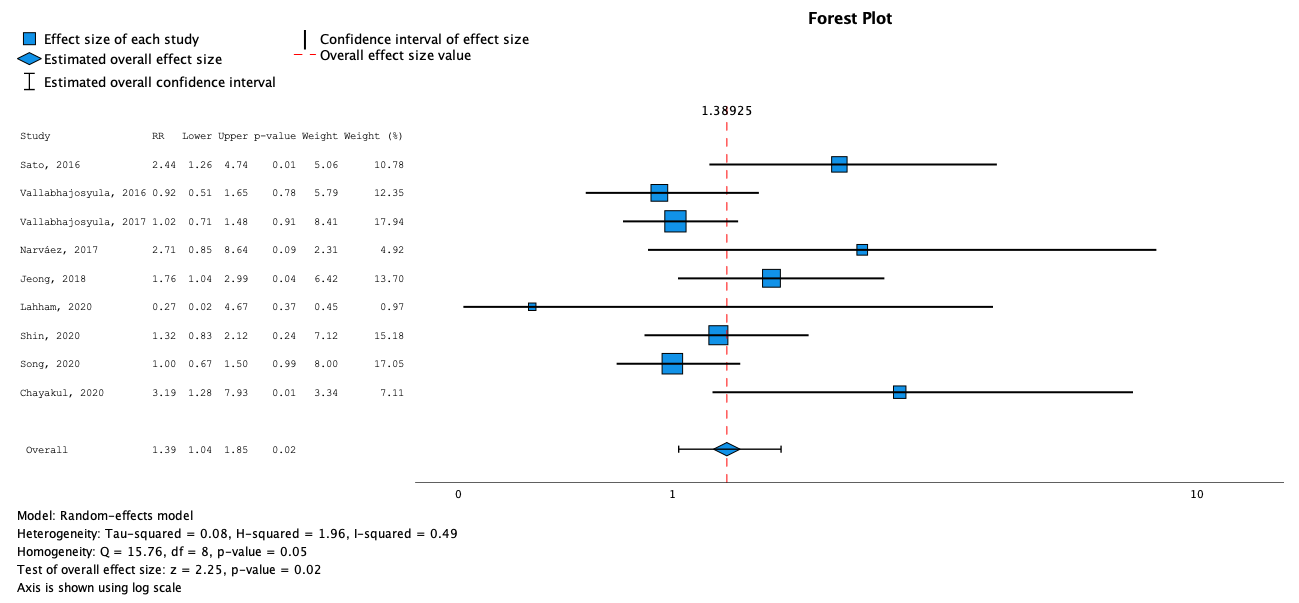


**Appendix Figure 5.** Subgroup analysis for in-hospital mortality. The selected studies were divided into sepsis diagnosis with sepsis II and sepsis III definitions. (1, sepsis II; 2, sepsis III)


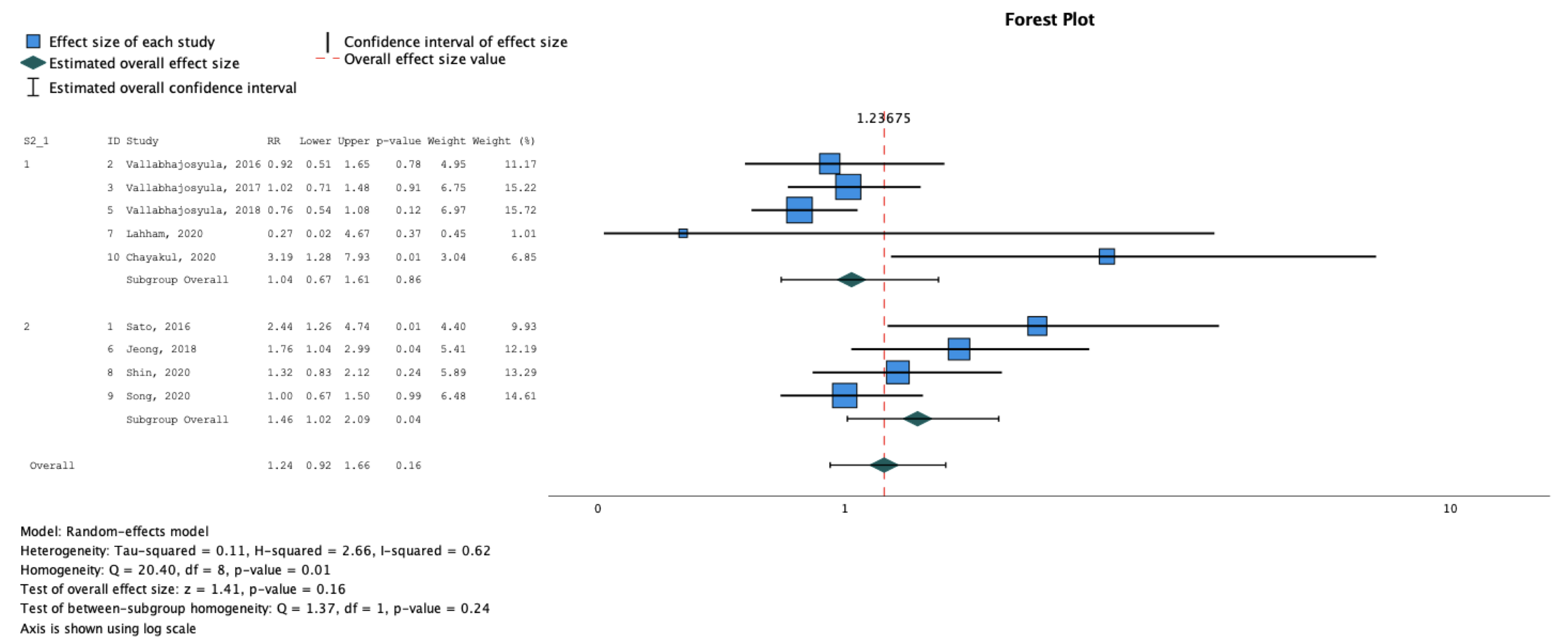


**Appendix Figure 6.** Subgroup analysis for in-hospital mortality. The selected studies were divided into Day-1, Day-2, Day-3 echocardiography screening. (1, Day 1; 2, Day 2; 3, Day 3)


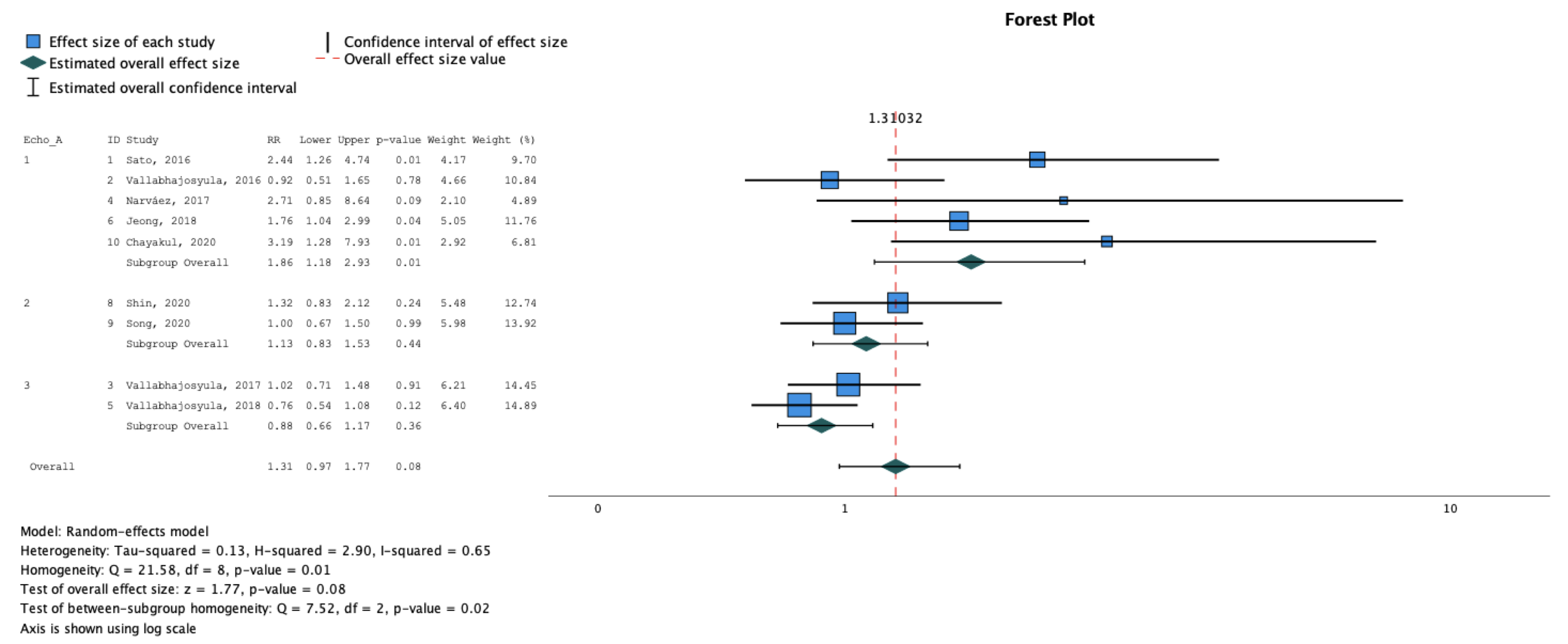


**Appendix Figure 7.** Subgroup analysis for in-hospital mortality. The selected studies were divided into echocardiography by protocol and by clinical needs. (1, by protocol; 2, by clinical needs)


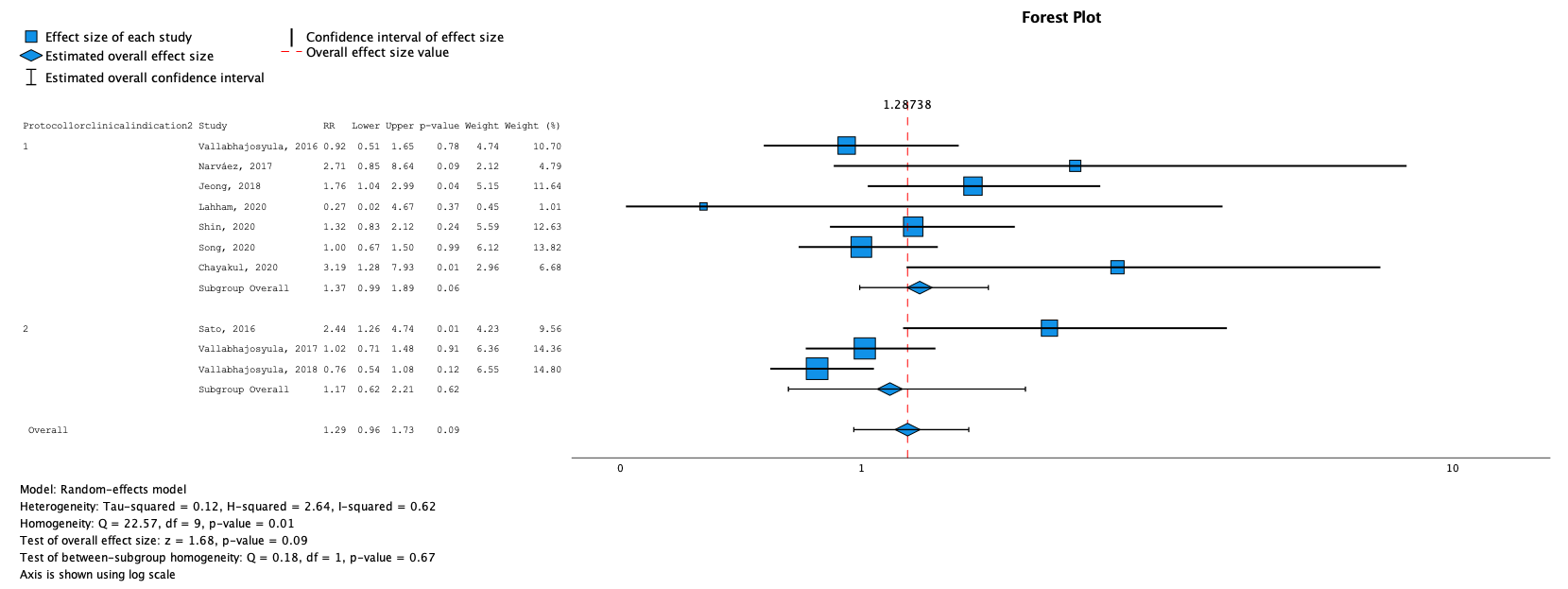


**Appendix Figure 8.** Subgroup analysis for in-hospital mortality. The selected studies were divided into the subgroups according to (1) left ventricular systolic dysfunction, (2) left ventricular diastolic dysfunction, (3) left ventricular dysfunction, (4) right ventricular dysfunction.


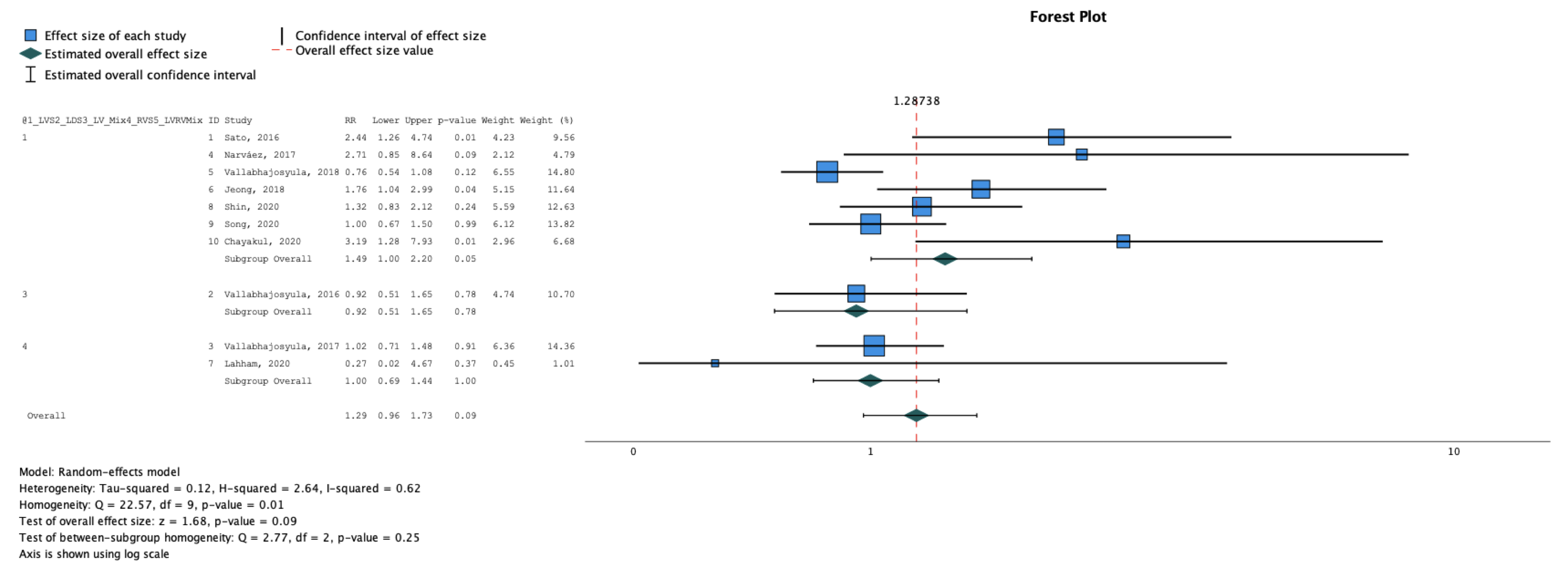


**Appendix Figure 9**. Subgroup analysis for in-hospital mortality. The selected studies were divided into the subgroups according to different cut-off values. 1. Only LVEF <50%, 2. LVEF<50% or LVEF reduction >10%, 3. LVEF<50%+E/e’>15, 4. RV S’ <15cm/s or TAPSE <16mm


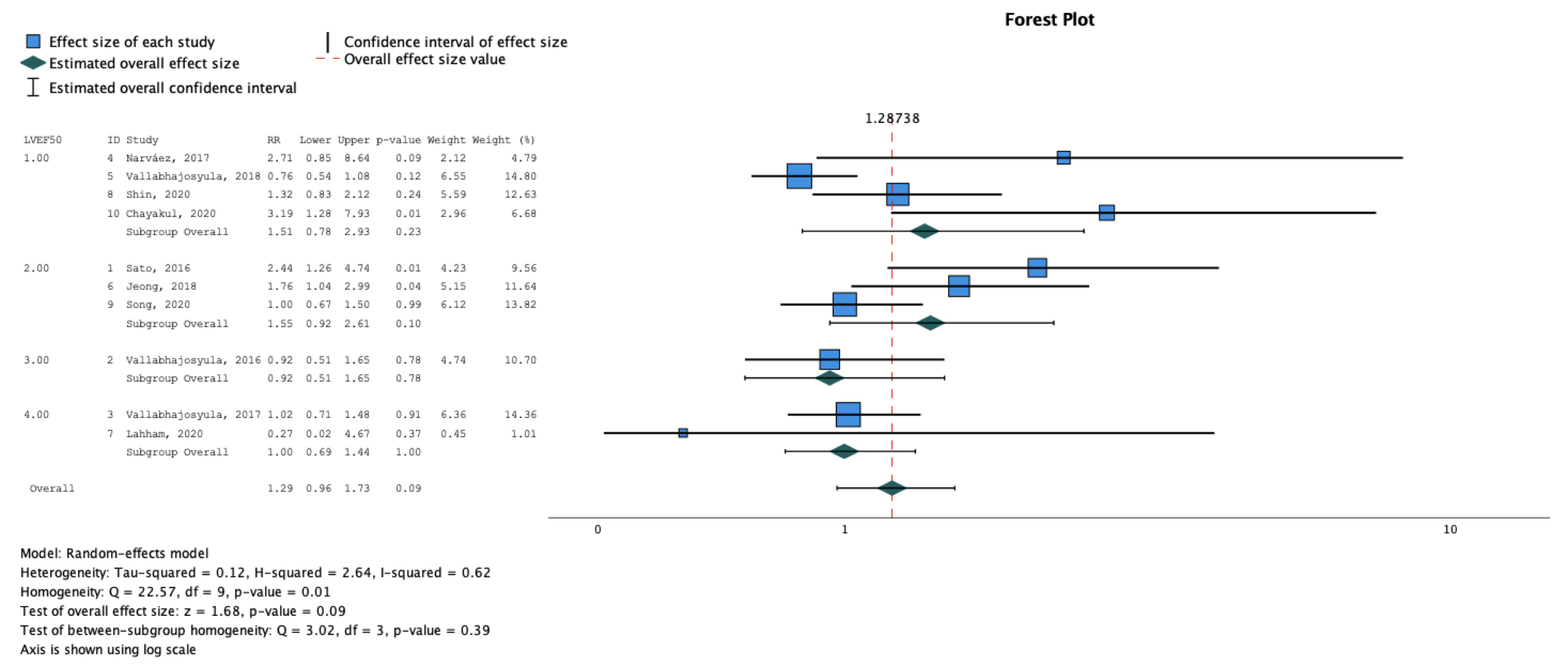


**Appendix Figure 10**. Subgroup analysis for in-hospital mortality. The selected studies were divided into the subgroups according to whether the risk estimate adjustment was performed in the selected studies. (1, without risk estimate adjustment; 2, with risk estimate adjustment)


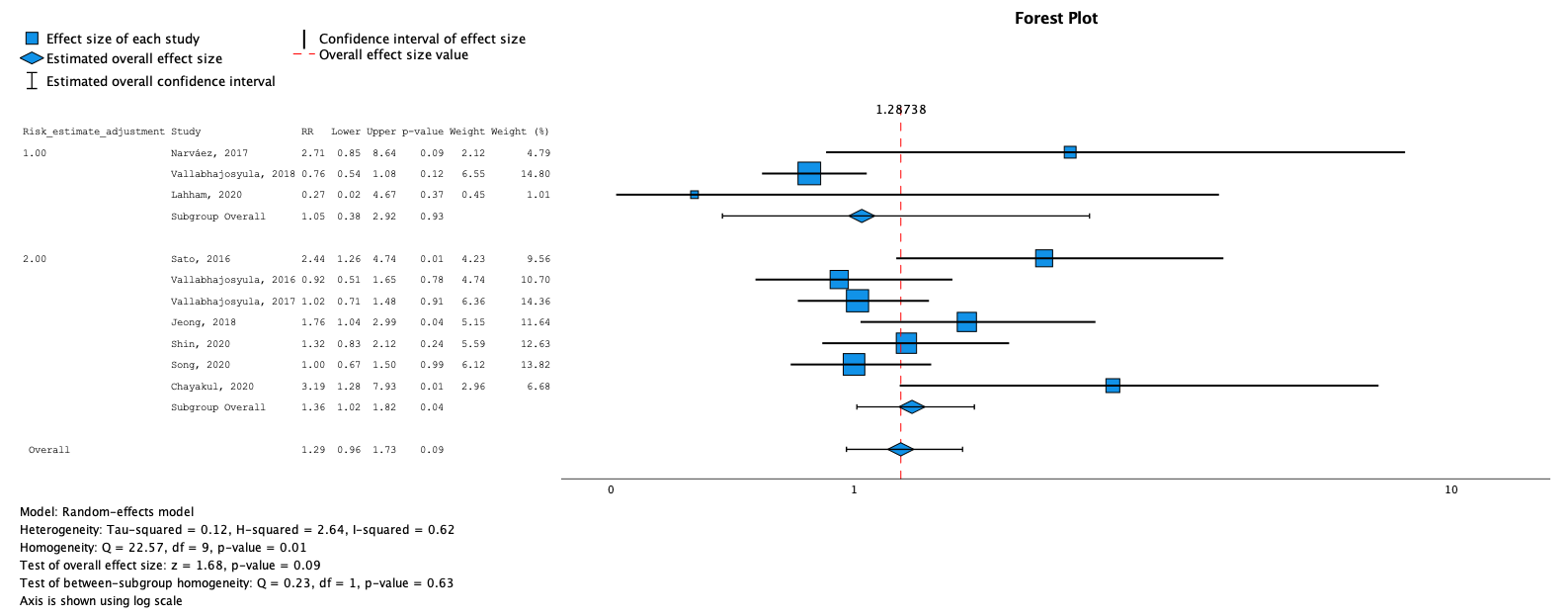


**Appendix Figure 11**. Subgroup analysis for in-hospital mortality. The selected studies were divided into the subgroups according to the appraisal quality of studies (1. Worse quality; 2. Better quality)


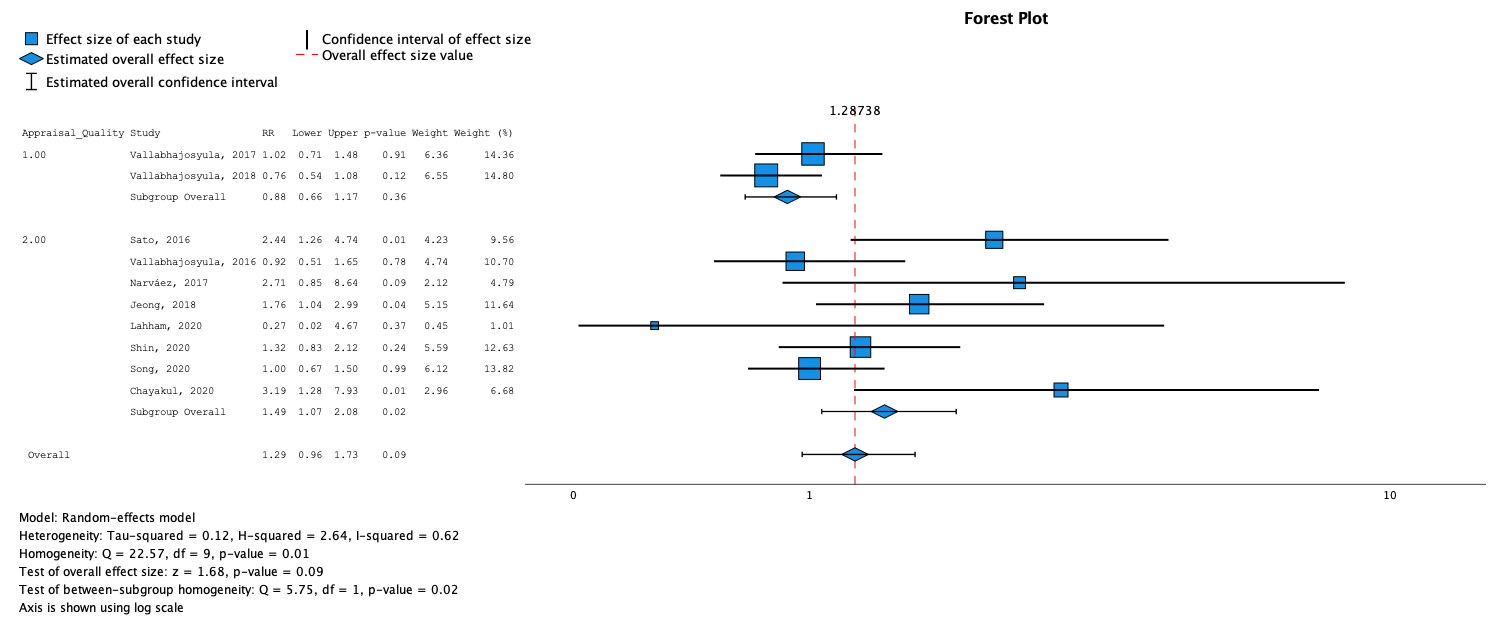


**Appendix Figure 12**. The bubble plot diagrams showed the meta-regression examining the heterogeneity in in-hospital mortality by different characteristics in the selected studies

**
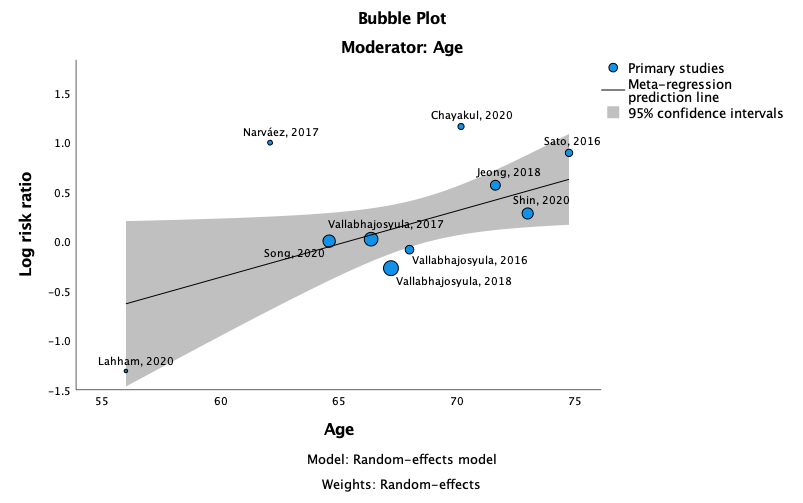
** **
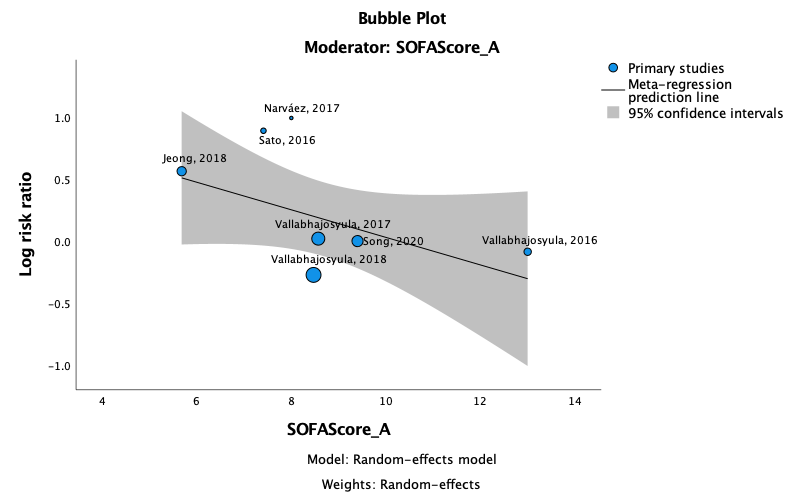
**

**
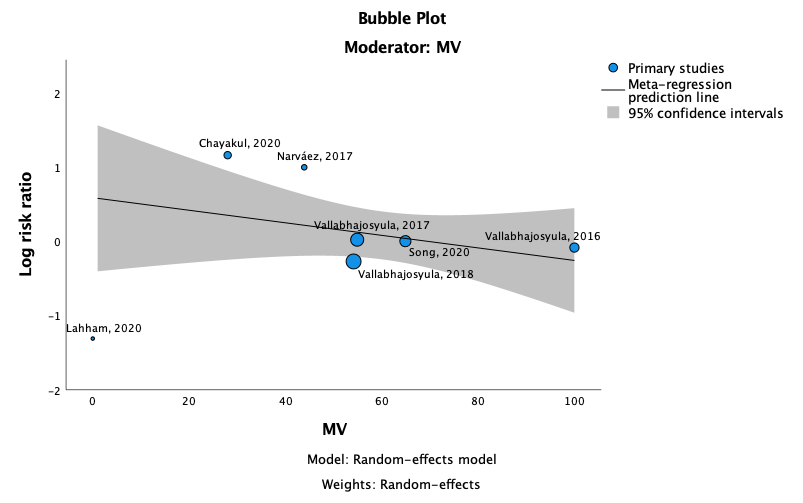
** **
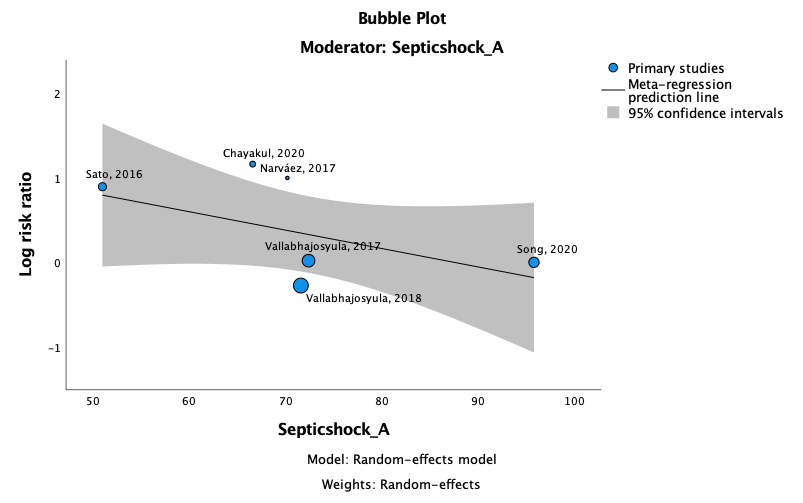
**

**Subgroup and sensitivity analyses for one-month mortality**

**Appendix Figure 13.** Sensitivity analyses of one-month mortality. Using the data for right ventricular dysfunction in Innocenti’s study


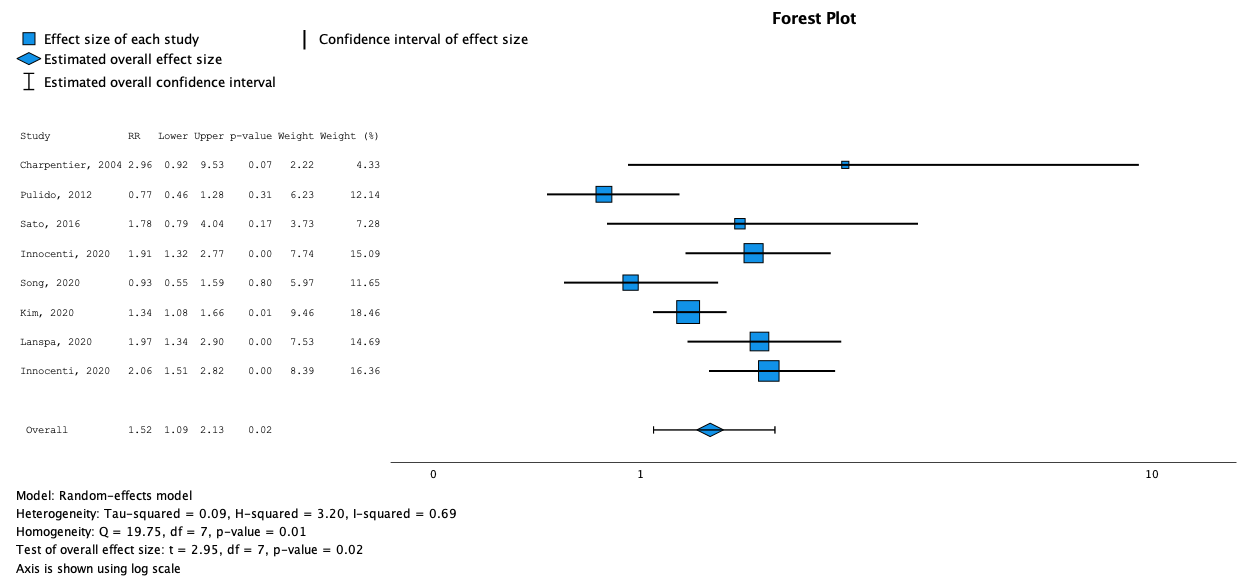


**Appendix Figure 14**. Sensitivity analyses of one-month mortality. Using the data for left and right ventricular dysfunction in Innocenti’s study Innocenti’s data (assumed no duplicated patients)


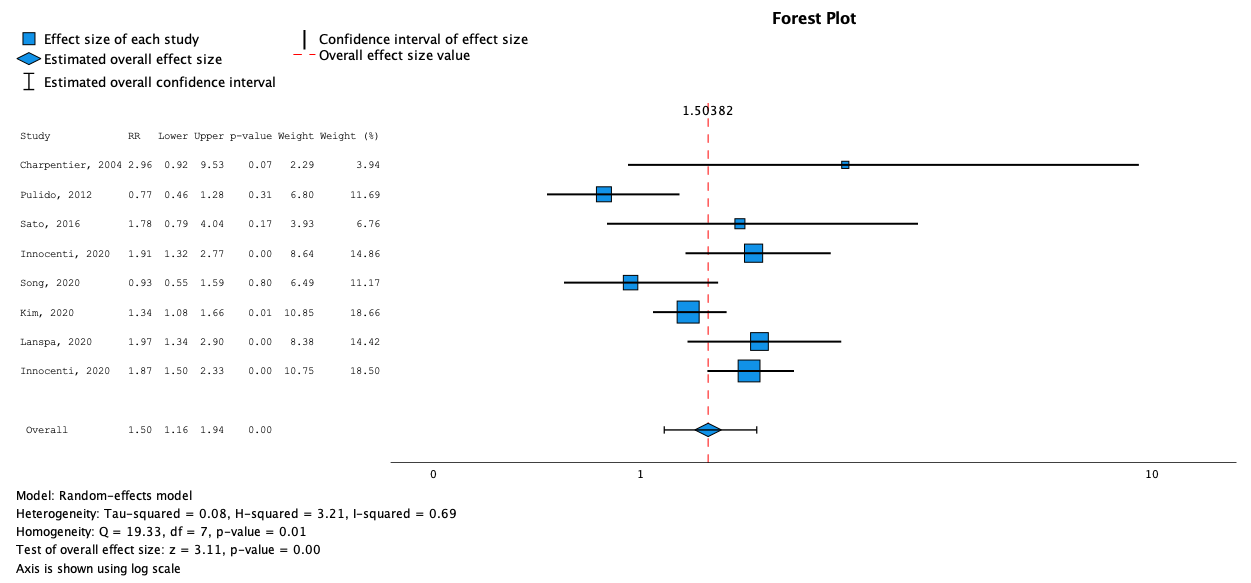


**Appendix Figure 15**. Sensitivity analysis for one-month mortality. Due to the possibility of duplicated patients, the sensitivity analysis excluded anyone study of Innocenti to evaluate the range of result uncertainty. (upper, excluding Innocenti, Mar 2020; lower, excluding Innocenti, Oct 2020)

**
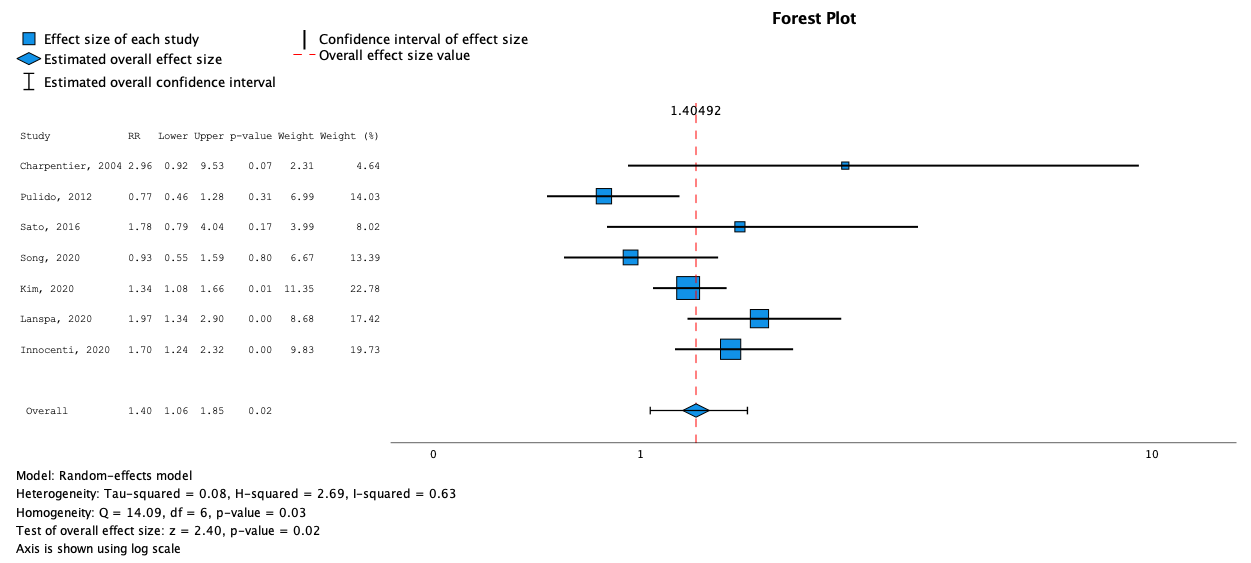
**


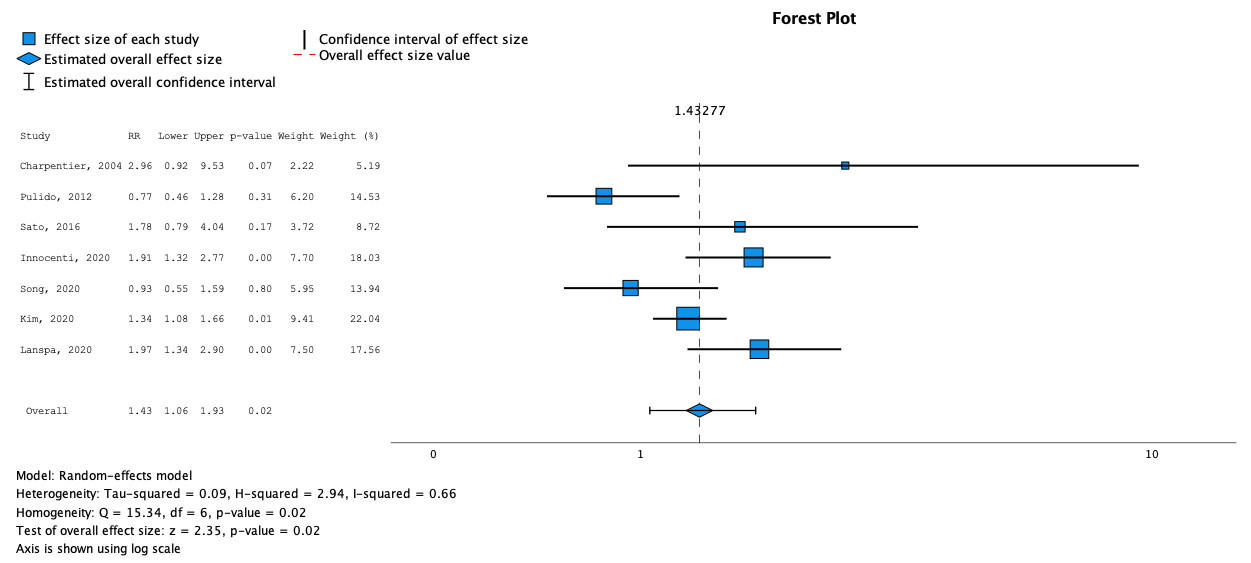


**Appendix Figure 16.** Subgroup analysis for one-month mortality. The selected studies were divided into sepsis diagnosis with sepsis II and sepsis III definitions. (1, sepsis II; 2, sepsis III)


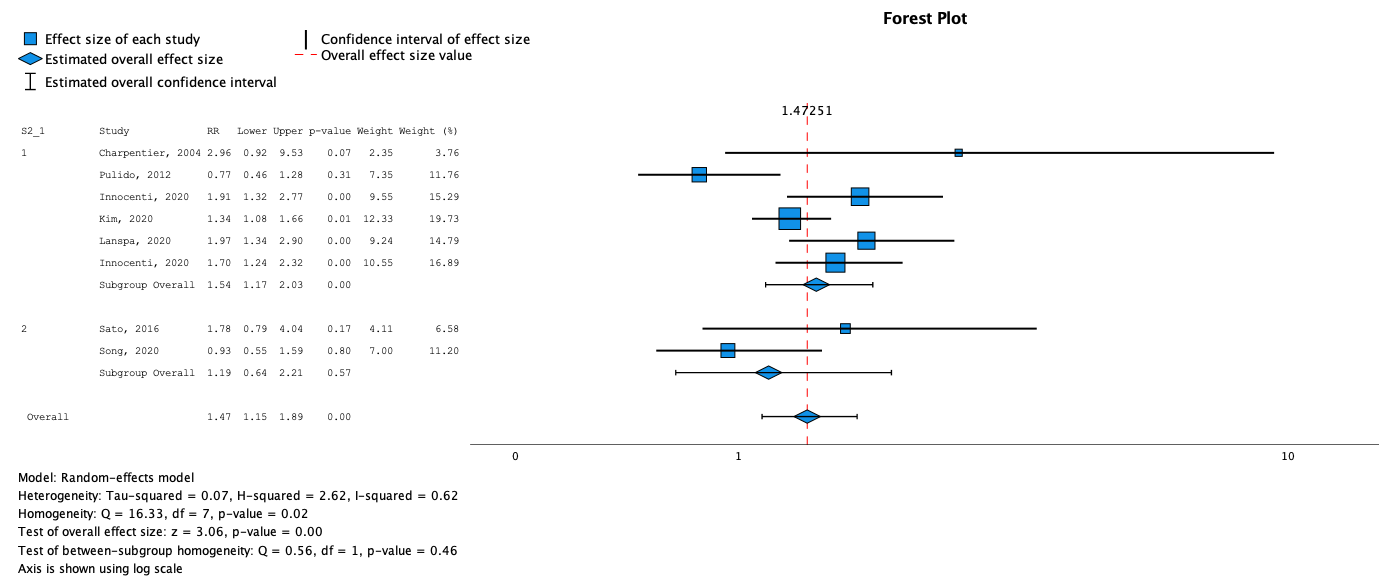


**Appendix Figure 17.** Subgroup analysis for one-month mortality. The selected studies were divided into Day-1, Day-2, Day-3 echocardiography screening. (1, Day-1; 2, Day-2; 3, Day-3)


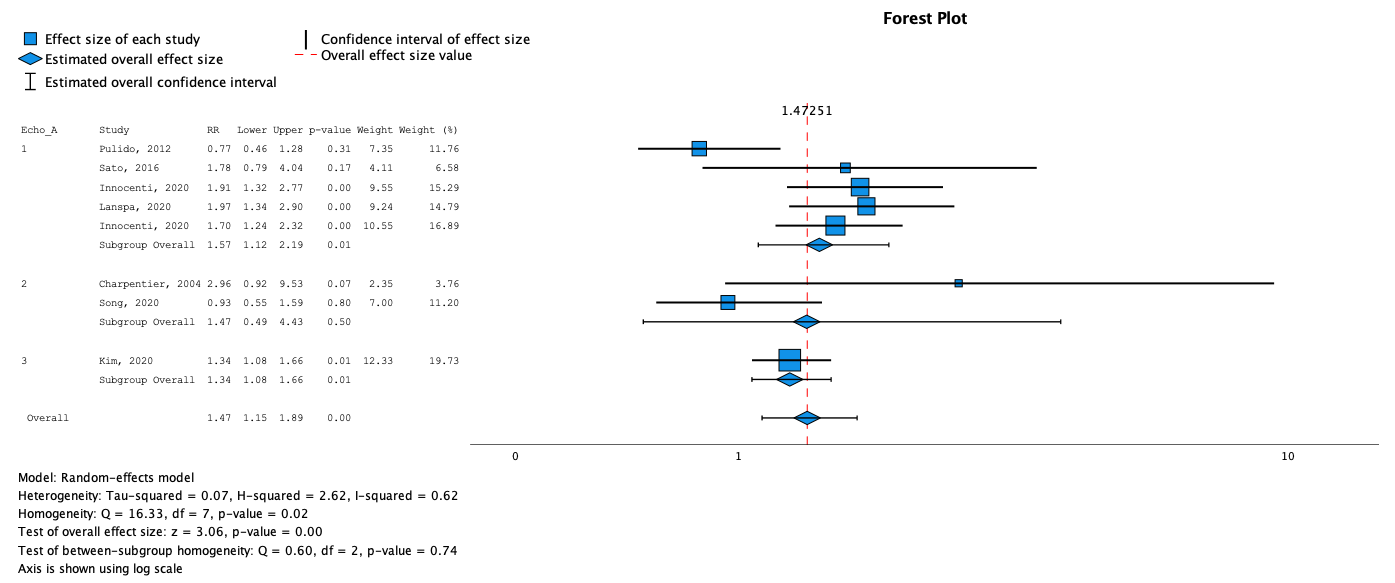


**Appendix Figure 18.** Subgroup analysis for one-month mortality. The selected studies were divided into echocardiography by protocol and by clinical needs. (1, by protocol; 2, by clinical needs)


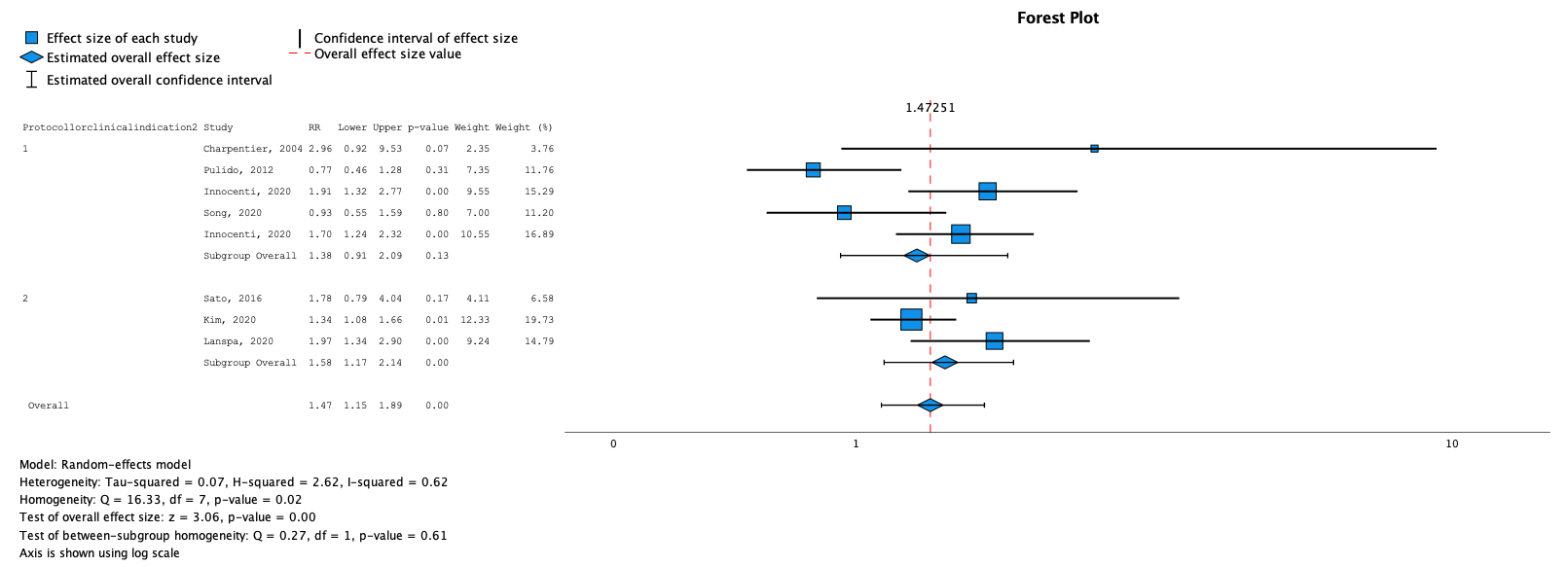


**Appendix Figure 19.** Subgroup analysis for one-month mortality. The selected studies were divided into the subgroups according to (1) left ventricular systolic dysfunction, (2) left ventricular diastolic dysfunction, (3) left ventricular dysfunction, (4) right ventricular dysfunction, (5) left and right ventricular dysfunction

**
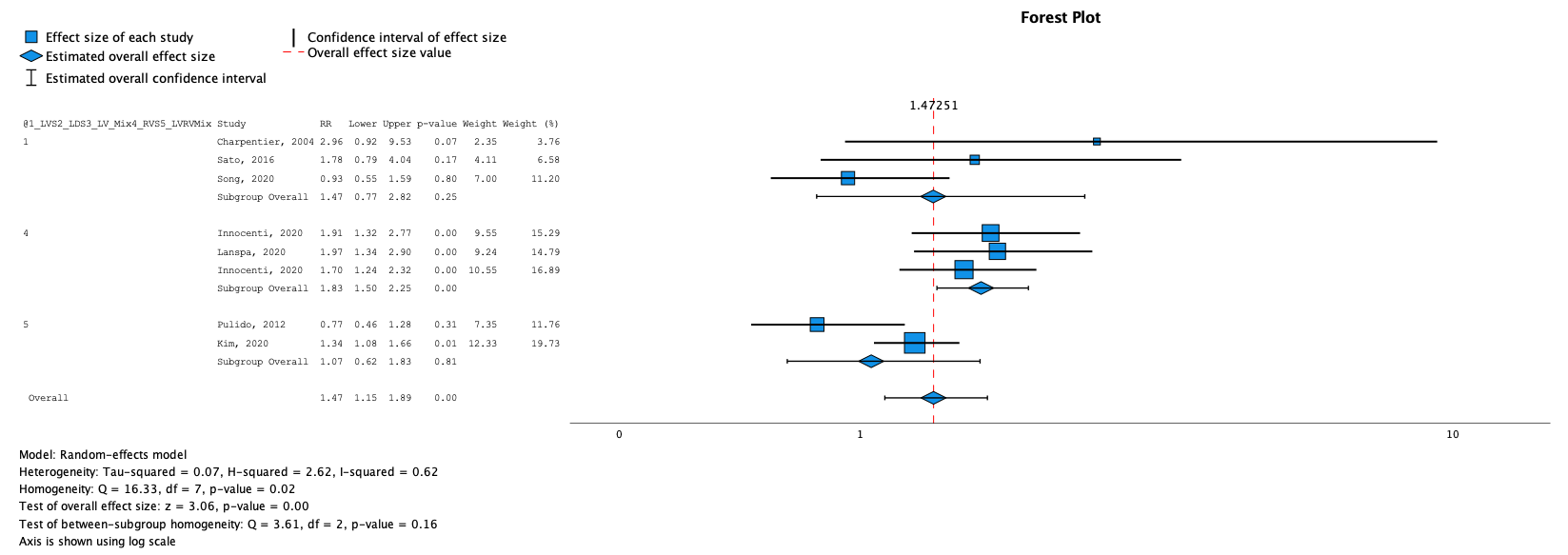
**

**Appendix Figure 20.** Subgroup analysis for one-month mortality. The selected studies were divided into the subgroups according to different cut-off values. 1. Only LVEF <50%, 2. LVEF<50% or LVEF reduction >10%, 3. LVEF<50%+E/e’>15, 4. RV S’ <15cm/s or TAPSE <16mm


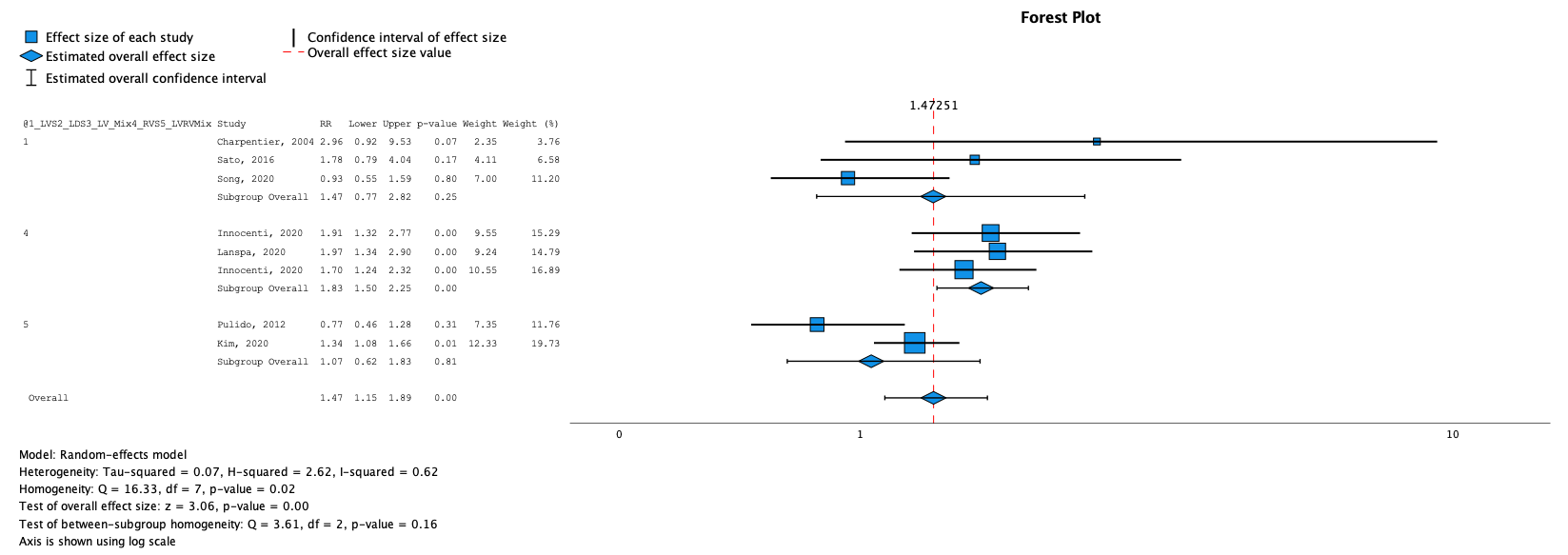


**Appendix Figure 21.** Subgroup analysis for one-month mortality. The selected studies were divided into the subgroups according to the appraisal quality of studies (1. Worse quality; 2. Better quality)


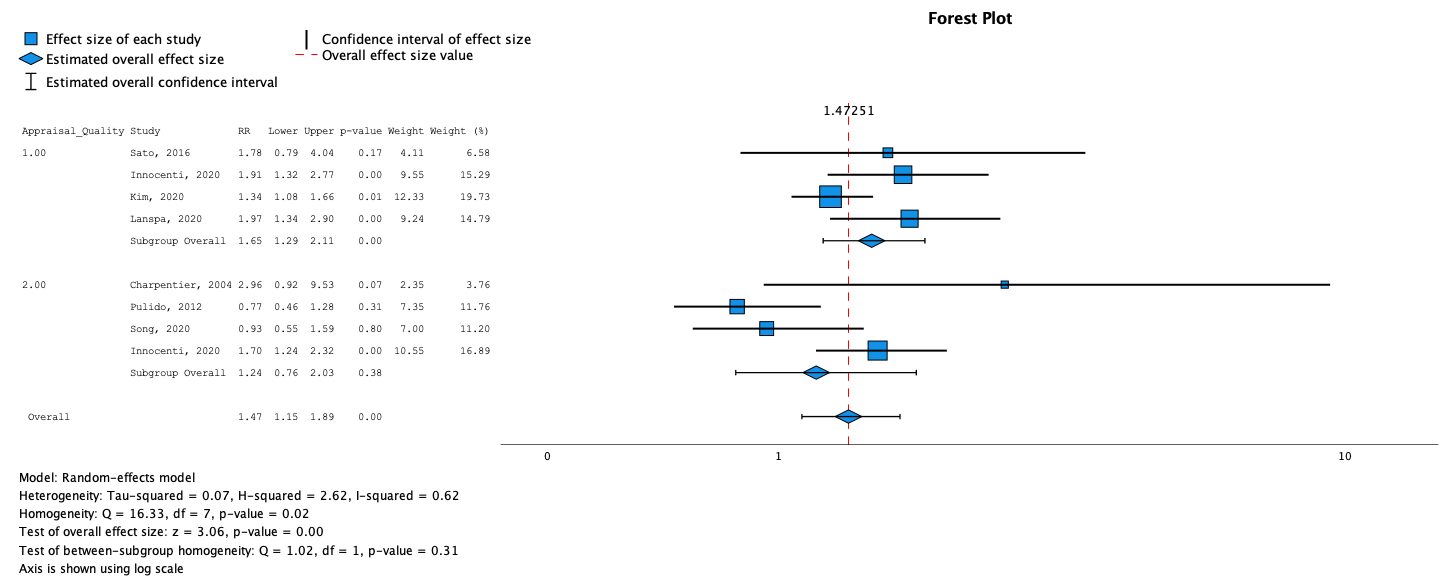


**Appendix Figure 22.** Subgroup analysis for one-month mortality. The selected studies were divided into the subgroups according to whether the risk estimate adjustment was performed in the selected studies. (1, without risk estimate adjustment; 2, with risk estimate adjustment)


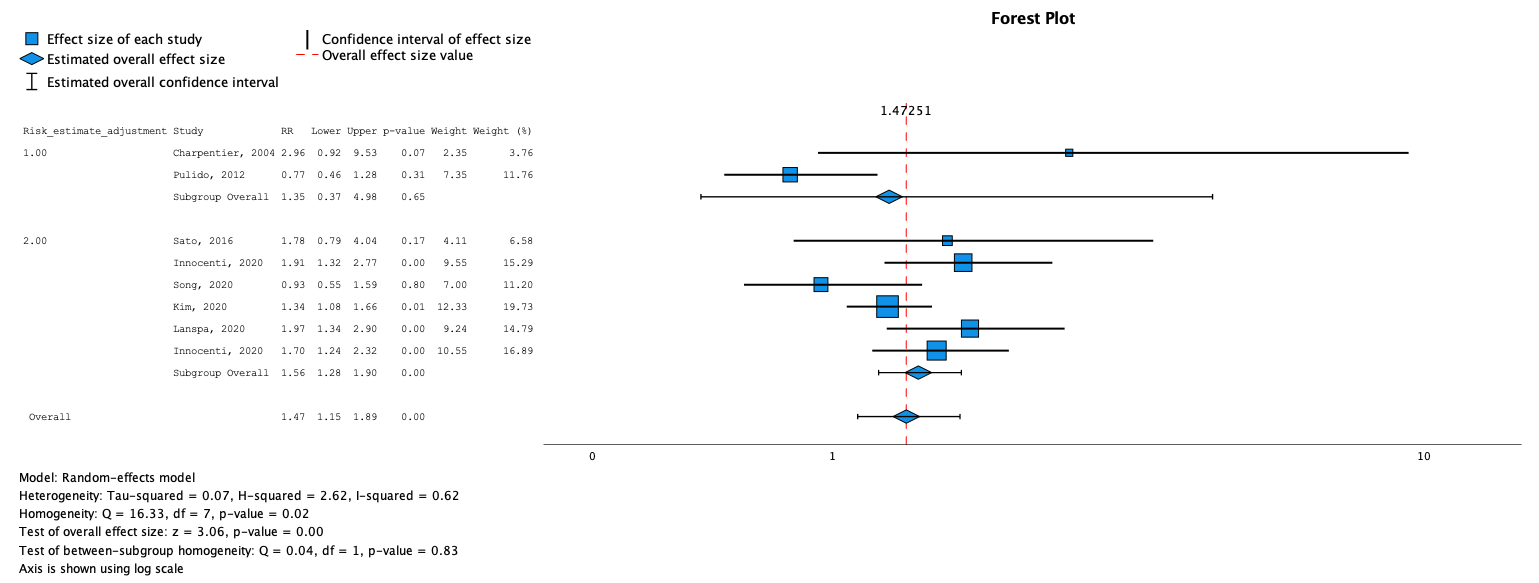


**Appendix Figure 23.** The bubble plot diagrams showed the meta-regression examining the heterogeneity in one-month mortality by different characteristics in the selected studies


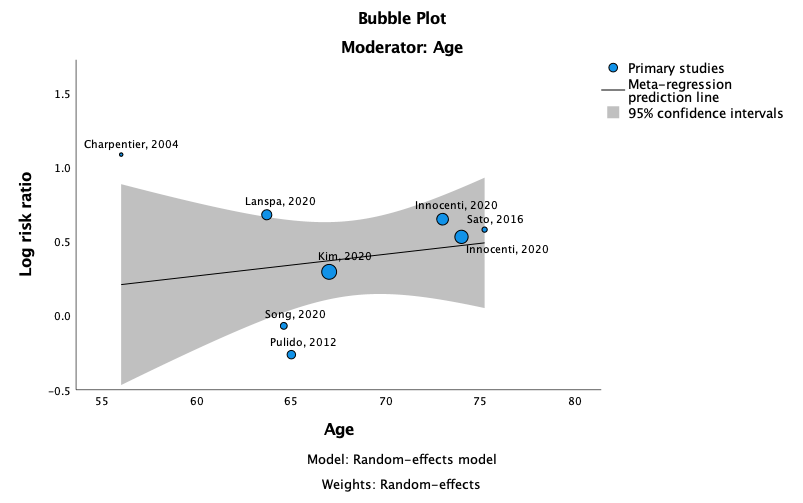

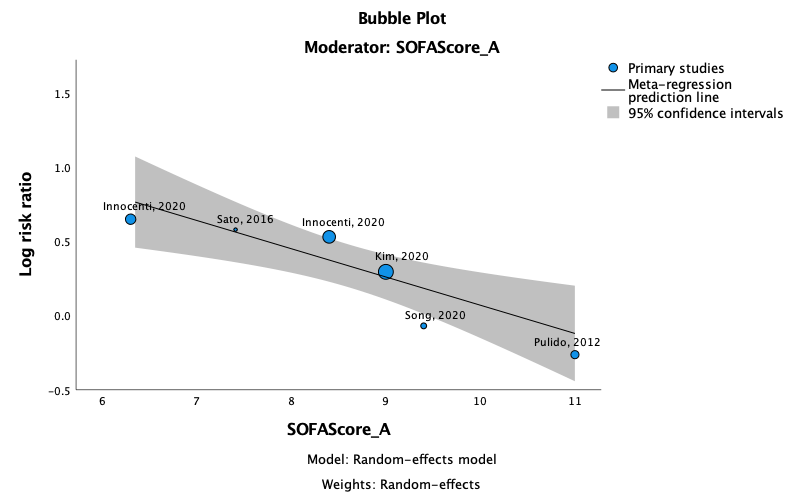


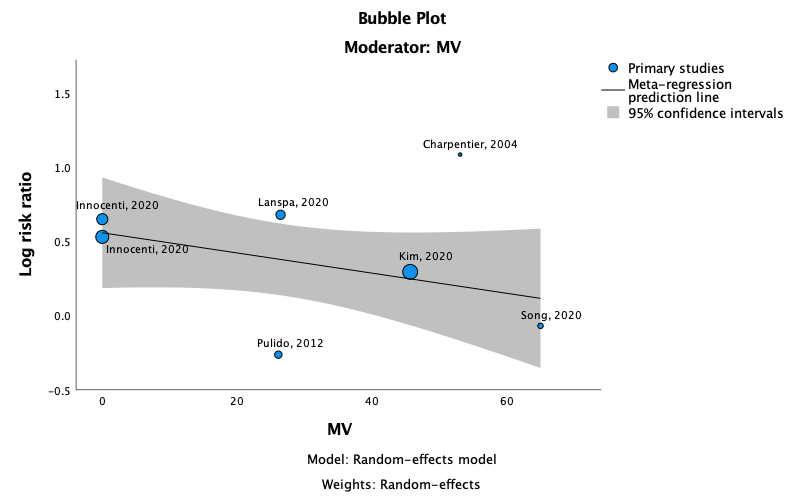

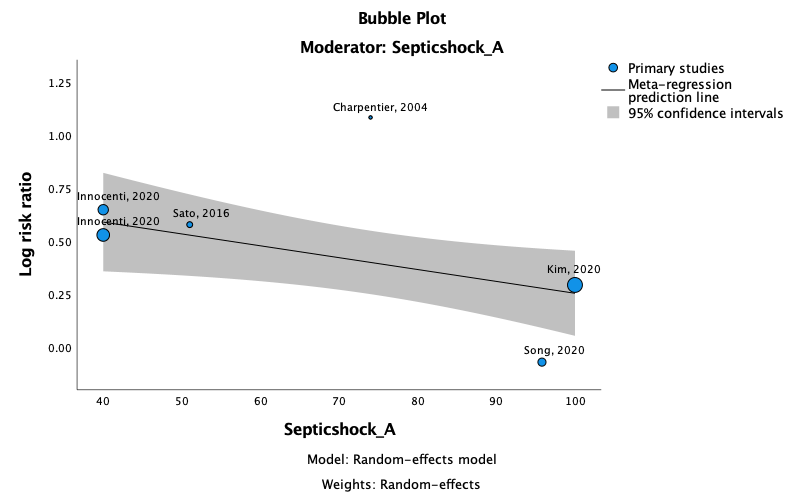

Supplement: Supplementary file 1 — Additional file 1: Appendix Table S1. Search strategy in PubMed and Embase on 8 July 2021. Appendix Table S2. Appraisal of cohort studies and case-control studies with Newcastle Ottawa Scale. Appendix Table S3. Risk estimates of the included studies. Appendix Table S4. Risk adjustment method of the included studies for data pooling. Appendix Table S5. One-by-one exclusion method for subgroup analysis of in-hospital mortality. Appendix Table S6. One-by-one exclusion method for sensitivity analysis of one-month mortality. Appendix Table S7. A random-effects meta-regress with Egger's regression-based test for the in-hospital mortality. Appendix Table S8. A random-effects meta-regress with Egger's regression-based test for the one-month mortality. Appendix Figure S1. The forest plot of the total selected studies 1. Mortality during ICU stay; 2. Mortality within 7 days; 3. Mortality within 10 days; 4. One-month mortality; 5. In-hospital morality; 6. One-year mortality; 7. Two-year mortality; 8. Mortality with non-defined duration. Appendix Figure S2. Sensitivity analyses of in-hospital mortality. Using the data for left ventricular diastolic dysfunction in Vallabhajosyula et al. 2016. Appendix Figure S3. Sensitivity analyses of in-hospital mortality. Using the data for left ventricular diastolic and diastolic dysfunction in Vallabhajosyula et al. 2016, with the assumption of no duplicated patients. Appendix Figure S4. Sensitivity analysis for in-hospital mortality. Due to the possibility of duplicated patients, the sensitivity analysis excluded anyone study of Vallabhajosyula to evaluate the range of result uncertainty. (upper, excluding Vallabhajosyula, 2017; lower, excluding Vallabhajosyula, 2018). Appendix Figure S5. Subgroup analysis for in-hospital mortality. The selected studies were divided into sepsis diagnosis with sepsis II and sepsis III definitions. (1, sepsis II; 2, sepsis III). Appendix Figure S6. Subgroup analysis for in-hospital mortality. The selected st [file 13613_2022_1089_MOESM1_ESM.docx]
